# Gene expression changes in mononuclear cells in patients with metabolic syndrome after acute intake of phenol-rich virgin olive oil

**DOI:** 10.1186/1471-2164-11-253

**Published:** 2010-04-20

**Authors:** Antonio Camargo, Juan Ruano, Juan M Fernandez, Laurence D Parnell, Anabel Jimenez, Monica Santos-Gonzalez, Carmen Marin, Pablo Perez-Martinez, Marino Uceda, Jose Lopez-Miranda, Francisco Perez-Jimenez

**Affiliations:** 1 Lipids and Atherosclerosis Research Unit. IMIBIC (Instituto Maimonides de Investigacion Biomedica de Cordoba), Reina Sofia University Hospital, University of Cordoba, and CIBER Fisiopatologia de la Obesidad y Nutricion, Spain; 2 Jean Mayer US Department of Agriculture Human Nutrition Research Center on Aging at Tufts University, Boston, Massachusetts, USA; 3 Department of Cell Biology, Physiology and Immunology, University of Cordoba, 14071 Cordoba, Spain; 4 IFAPA Centro Venta del Llano, Junta de Andalucía, P.O. Box 50, Mengibar, Jaen E-23620, Spain

## Abstract

**Background:**

Previous studies have shown that acute intake of high-phenol virgin olive oil reduces pro-inflammatory, pro-oxidant and pro-thrombotic markers compared with low phenols virgin olive oil, but it still remains unclear whether effects attributed to its phenolic fraction are exerted at transcriptional level *in vivo*. To achieve this goal, we aimed at identifying expression changes in genes which could be mediated by virgin olive oil phenol compounds in the human.

**Results:**

Postprandial gene expression microarray analysis was performed on peripheral blood mononuclear cells during postprandial period. Two virgin olive oil-based breakfasts with high (398 ppm) and low (70 ppm) content of phenolic compounds were administered to 20 patients suffering from metabolic syndrome following a double-blinded, randomized, crossover design. To eliminate the potential effect that might exist in their usual dietary habits, all subjects followed a similar low-fat, carbohydrate rich diet during the study period. Microarray analysis identified 98 differentially expressed genes (79 underexpressed and 19 overexpressed) when comparing the intake of phenol-rich olive oil with low-phenol olive oil. Many of these genes seem linked to obesity, dyslipemia and type 2 diabetes mellitus. Among these, several genes seem involved in inflammatory processes mediated by transcription factor NF-κB, activator protein-1 transcription factor complex AP-1, cytokines, mitogen-activated protein kinases MAPKs or arachidonic acid pathways.

**Conclusion:**

This study shows that intake of virgin olive oil based breakfast, which is rich in phenol compounds is able to repress *in vivo *expression of several pro-inflammatory genes, thereby switching activity of peripheral blood mononuclear cells to a less deleterious inflammatory profile. These results provide at least a partial molecular basis for reduced risk of cardiovascular disease observed in Mediterranean countries, where virgin olive oil represents a main source of dietary fat. Admittedly, other lifestyle factors are also likely to contribute to lowered risk of cardiovascular disease in this region.

## Background

Metabolic syndrome (MetS) is a very common condition that is associated with increased cardiovascular disease (CVD) risk and type 2 diabetes mellitus, itself a risk factor for CVD. Its diverse clinical characteristics illustrate the complexity of the disease, involving several dysregulated metabolic pathways and multiple genetic targets. When caloric intake exceeds caloric expenditure, a positive caloric balance and subsequent storage of fat in adipose tissue appears; this often is cause of adipocyte hypertrophy and obesity. Depending on interaction of genetic and environmental factors, this hypertrophy can be followed by macrophage accumulation within adipose tissue leading to local hypoxia, inflammation, and oxidative stress. These deleterious effects favor adipose functional failure resulting in changes in systemic energy delivery; these also impair glucose consumption and activate self-regulatory mechanisms that extert influence over whole body homeostatic systems and link obesity to numerous health problems associated with MetS [1]. A low-level systemic chronic inflammation, with abnormal cytokine production in adipose tissue, increased acute-phase reactants, and activation of inflammatory signaling pathways, often acting over a period of many years, appears to constitute a potential link to atherosclerosis development in these patients [2]. In addition, changes in postprandial metabolism undergoing every time we have a meal as well as alterations in this state may play an important role in the development of cardiovascular and cardiovascular associated diseases [3]. Moreover, Van Oostromet et al. [4] provides evidence that postprandial triglyceridemia is related to a pro-inflammatory state due to high expression of markers of neutrophilic and monocyte activation.

The anti-atherogenic effects associated with Mediterranean Diet (MD) rich in Virgin Olive Oil (VOO) consumption, could contribute in explaining the low rates of cardiovascular mortality found in Southern European Mediterranean countries, in comparison with other Western populations [5]. It has been demonstrated recently that inhibition of circulating immune cell activation could be a protective mechanism by which MD exerts its healthy effects [6]. Bonani et al. [7] showed that extra VOO consumption reduces inflammatory markers and increase serum antioxidant capacity at postprandial state. Previously, our group has showed how a VOO-rich MD, during postprandial state, reduces inflammatory response of peripheral blood mononuclear cells (PBMCs) mediated by transcription factor NF-κB, when compared to, butter and walnut-enriched diets or Western diets [8,9]. These results have been replicated by others and support the hypothesis that decreasing NF-κB pathway activation is a mechanism involved in the anti-inflammatory effect of a VOO-rich MD [10,11]. Nevertheless, it has been proposed that healthy effects of VOO may be due not only to its oleic acid content but also to the antioxidant and anti-inflammatory capacity of minor components, especially the phenol-rich fraction [12,13]. Our group demonstrated that the VOO phenol fraction also improves endothelial dysfunction and haemostatic profile during postprandial state [14,15]. Other studies showed how these compounds ameliorate lipid profiles and decrease oxidative stress *in vivo *[12]. However, in animal models, it has been showed that hydroxytyrosol, a phenol found in olive oil, administered after being extracted from its original matrix could be not only non beneficial but indeed harmful for health [16].

It has been speculated that potentially beneficial effects could be due to olive oil modulation on genes involved in proliferative, antioxidant and inflammatory pathways. In order to clarify this question, some human, animal models and *in vitro *studies were undertaken using gene expression microarray platforms [17]. These studies showed that olive oil is able to modify gene expression coding for proteins participating in cellular mechanisms involved in oxidative stress resistance [18], lipid metabolism [19] and other atherosclerosis-related traits/pathways [20,21]. Nonetheless, it is unresolved whether these changes in gene expression are mediated by oleic acid or executed by olive oil polar minor components, maybe as a consequence of their antioxidant capacity, or by interacting directly with receptors, enzymes or transcription factors. To date, no intervention studies in humans have thoroughly explored the effects of VOO phenol compounds on gene expression. Thus, the main purpose of this study was to identify genes which undergo changes in expression in PBMCs in patients with MetS, after acute intake of breakfast based in phenol-rich virgin olive oil, compared to phenol-poor olive oil breakfast.

## Results

### Postprandial metabolic parameters

No significant differences were observed in any of the iAUCs of the main metabolic variables (glucose, insulin, non-sterified fatty acids, serum triglycerides and high density lipoprotein cholesterol) after intake of phenol-rich virgin olive oil compared with olive oil with lower content of these compounds (Table 1).

**Table 1:** Postprandial metabolic parameters.

	Low-phenol OO breakfast	High-phenol OO breakfast	p value
iAUC Glucose (mg.dL-1. min-1)	1775 ± 5156	1556 ± 2661	0.876
iAUC Insulin (U.L-1.min-1)	3722 ± 2904	4141 ± 2239	0.627
iAUC NEFA (mmol.L-1.min-1)	-4257 ± 3321	-4029 ± 2626	0.990
iAUC TG (mg.dL-1. min-1)	4256 ± 3504	4277 ± 6441	0.995
iAUC HDLc (mg.dL-1. min-1)	112 ± 689	135 ± 1036	0.395

Incremental area under the postprandial serum concentration curve (iAUC) of glucose, insulin, NEFA, TG and HDLc after the acute intake of both types of olive oil. OO, olive oil; p values correspond to Wilcoxon-paired test (n = 20); iAUC, incremental area under the concentration curve; NEFA, non-sterified fatty acids; TG, serum triglycerides; HDLc, high density lipoprotein cholesterol. All values are expressed as mean ± SD.

### Microarray results and selection of candidate genes

Two color microarrays experiments were performed by using Agilent platforms; 45,220 probe sets were tested to interrogate the expression of 30,886 unique human genes. Microarray analysis showed a correlation index >90% of the raw log-intensity signal among replicates in the array in all cases. Mean coefficient of variation of the log-signal probe values was lower than 0.1 for intra-array replicates. Changes in gene expression were determined as log_2 _ratio of signal intensity values corresponding to transcripts present 4 hours after intake of olive oil with high phenol content, divided by the signal intensity values, which correspond to transcripts present 4 hours after consumption of low phenol content olive oil. 15,308 high-quality probes were selected ranging from 1.89 to -1.79 log_2 _ratio. In these, we found 98 genes differentially expressed in human PBMCs (Additional File 1), selecting log_2 _ratio greater than 0.4 (more expression after intake of olive oil with high phenol content, this is, at least 1.32 fold, in 19 over-expressed genes) or log_2 _ratio lower than -0.4 (less expressed after consumption of high phenol content olive oil; this is, at least 1.32 fold, in 79 under-expressed genes) with a statistical significance (*p*) for the fitted linear model of = 0.01. Results were adjusted by dye-swap effects and by False Discovery Rate (FDR) using the Benjamini and Hochberg method. The most under-expressed genes in MetS patients during postprandial period after intake of olive oil phenols were *G0S2, EGR2, EGR1, FOSB, IL1B, NR4A2, EGR3, RASGEF1B, CXCL1 and PTGS2 *(1.95- to 2.74 fold) and the most over-expressed genes were CA1, RAP1GAP, GYPB, FN1 and SELENBP1 (1.46- to 1.57- fold). Additionally, we performed a gender analysis (Additional File 2). From these, we found 32 genes differentially expressed in the analysis for both, men and women. However, 329 genes were differentially expressed only for men (218 genes) and only for women (111 genes).

### Results validation by qRT-PCR

**Figure 1:**
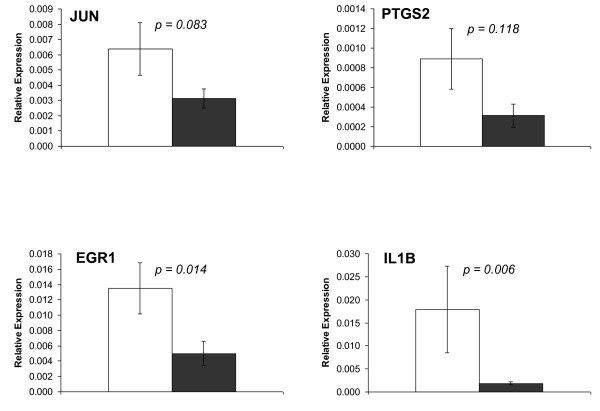
**Microarrays results validation by qRT-PCR**. Mean ( ± S.E.M.) of relative expression values after the consumption of low and high phenol content olive oil. One way ANOVA p value is showed. Gene expression values were log transformed before statistical analyses.

**Figure 2:**
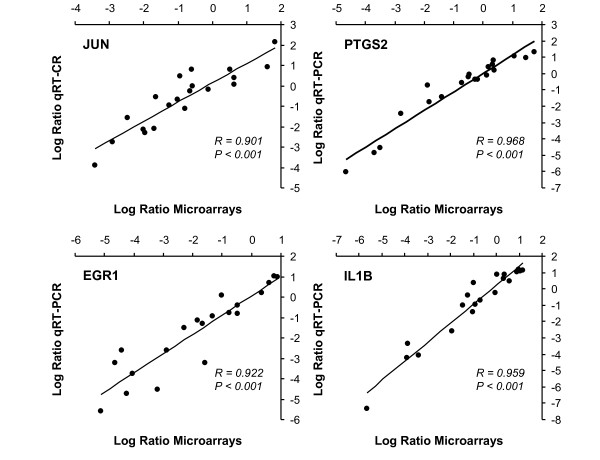
**Correlation between microarrays and qRT-PCR results**. Correlation analysis between log_2 _ratio of the gene expression values from microarrays and qRT-PCR experiments. Ratio of the gene expression values correspond to gene expression after consumption of high phenol content olive oil divided by gene expression after the consumption of low phenol content olive oil. Spearman r values are shown in the graphs.

To confirm microarray results using an independent technique, log_2 _of the normalized microarray ratio values were compared with log_2 _ratio values for four genes obtained from qRT-PCR experiments (*JUN*, *PTGS2*, *EGR1 *and *IL1B*). Results showed that features of the changes in both procedures were similar with Spearman r coefficients between 0.901 and 0.968 and *p*-values less than 0.001 (Figure 1 and 2).

### Pathway analysis

**Figure 3:**
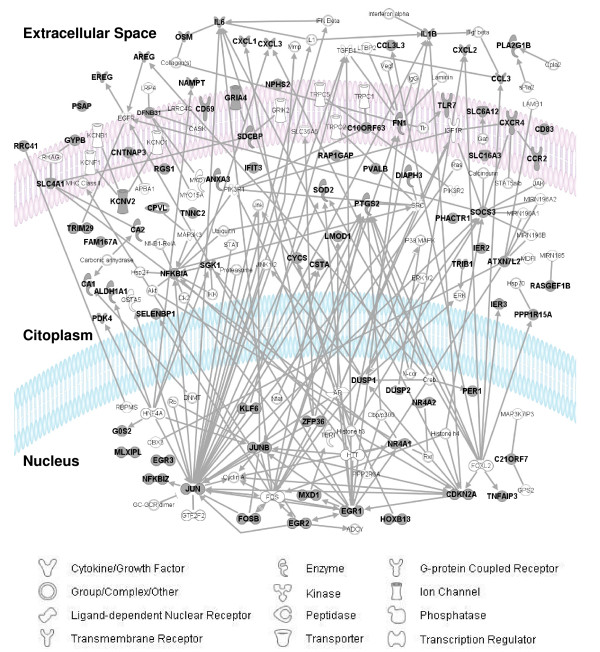
**Ingenuity Pathway Analysis Network**. Network of phenol-rich VOO modulated genes. Gray symbols denote that the gene was found over-expressed or under-expressed by phenols in microarrays analysis.

In order to investigate functional relationships in the set of differentially expressed genes, we used the Ingenuity Pathway Analysis Software [22] (Ingenuity Systems, Redwood City, CA USA) which employs a predefined knowledge base containing over 10.000 curated human genes. Of the 98 differentially expressed genes found in patients with MetS during the postprandial period after intake of olive oil phenols, two transcripts (LOC284454, AF351612) showed no entries within the knowledge base and only 81 genes were eligible for network analysis. Inflammatory disorder was the most highly represented disorder (39 genes, *p *= 2.11E-19). From these, 35 genes (*PTGS2, IL1B, IL6, OSM, CCL3, CXCL1, CXCL2, CXCL3, CXCR4, NAMPT, DUSP1, DUSP2, EGR1, EGR2, EGR3, EREG, FOSB, G0S2, JUN, JUNB, NFKBIA, NFKBIZ, NR4A1, NR4A2, PER1, SOCS3, SOD2, TAGAP, TNFAIP3, ZFP36, AREG, CA2, CD69, CD83, CDKN2A*) were under-expressed and after intake of virgin olive oil with high content in phenolic compounds, 4 genes were over-expressed (CCR2, *CA1, CPVL, FN1*). Cellular functions most strongly associated with the differentially expressed genes were cell death (41 genes, *p *= 7.53E-11), cell migration (24 genes, *p *= 3.88E-8), cell division (23 genes, *p *= 6.29E-8), cell proliferation (32 genes, *p *= 9.90E-7) and transcription (25 genes, *p *= 3.55E-05). Network analysis yielded 14 different sub-networks, from which, one was identified as preferentially enriched by genes found differentially expressed after high-phenol VOO intake. This top-scoring sub-network included 26 genes whose related top-function was 'inflammatory diseases' with a probability value of 10^-54 ^(Fisher's exact test) of gene interrelationships being by chance. All sub-networks were merged to obtain the overall network shown in Figure 3. Interacting proteins were added using Ingenuity Pathways Knowledge Base database. Finally non-connected genes or those connected by two or more edges were removed, except for those genes that were found to be differentially expressed in our microarrays analysis.

## Discussion

In the present study, we have observed that the phenol fraction of VOO *in vivo *is able to repress the expression of several genes related to inflammation pathways in patients with MetS during postprandial period (Additional File 1). This finding draws interest since pro-inflammatory state remains as one component of MetS [23] and low-grade inflammation is often associated with endothelial dysfunction [24], which by itself is associated to the development of atherosclerosis [25].

The *PTGS2 *gene encodes prostaglandin-endoperoxide synthase 2 (COX-2), an inducible isozyme involved in prostaglandin biosynthesis using arachidonic acid as substrate. In macrophages, and other cells, COX-2 activity is rapidly increased by various stimuli, such as pro-inflammatory cytokine IL1β. Substantial evidence indicates that up regulated *PTGS2 *expression and prostaglandin synthesis indeed influence chronic inflammatory conditions [26]. De la Puerta et al. observed that murine macrophages showed significantly reduced IL1β production and COX-2 activity after olive oil-enriched diet [27]. Recently, it has been demonstrated that hydroxytyrosol, one of the most important phenol compounds found in virgin olive oil, attenuates *in vitro *LPS-induced transcription of *PTGS2 *[28]. Our study showed that *in vivo *intake of phenol-rich virgin olive oil in humans is associated with a decreased expression of both *IL1B *and *PTGS2*, as compared to low-phenol olive oil intake. Those effects could contribute to reduced inflammation during postprandial state in agreement with anti-inflammatory effects observed after VOO-rich MD consumption [8,9].

The cytokine-cytokine interaction pathway contains a network of proteins (chemokines and their receptors) involved in the recruitment and activation of leukocytes during inflammatory response. Expression of genes such as *CCL3*, *CXCL1*, *CXCL2*, *CXCL3*, *CXCR4*, *IL1B*, *IL6*, and *OSM *is described under-expressed after acute intake of phenol-rich olive oil in our intervention study on diet. *CCL3 gene*, which codes for macrophage inflammatory protein-1 (MIP-1), has been implicated in monocyte infiltration of adipose tissue, an action that could significantly influence a pro-inflammatory pattern within endothelial cells [29]. *CXCL1*, *CXCL2 *(*MIP2A*), and further, *CXCL3 *(*MIP2B*) are genes for small and structurally related chemokines that regulate cell trafficking of various types of leukocytes through interactions with a subset of G protein-coupled receptors. Elgazar-Carmon et al. have demonstrated in mice that early neutrophil infiltration of adipose tissue may be mediated by *CXCL1*, a process that would precede macrophage infiltration after long-term consumption of a high-fat diet [30]. *IL6 *encodes a cytokine which is secreted to serum and induces a transcriptional response involved in a wide variety of inflammation-associated conditions, including MetS and type 2 diabetes mellitus (T2DM) [31]. On the other hand, it has been proposed that *IL6 *and *OSM*, which encodes oncostatin M, a growth regulator and member of the *IL6 *group of cytokines, can contribute to the increased cardiovascular risk in obese patients by up regulating PAI -1 in adipose tissue [32].

Activation of NF-κB and MAPK pathway, a cascade of phosphorilation events that result in the activation of transcription factors like CREB and AP-1, synergize for expression of inflammatory genes through coordinate bindings of transcription factors to κB and AP-1 sites which have been found together in the promoters of e.g. IL6 and TNF-α, and many other inflammatory genes [33]. Chemokine repression found in our study could be consequence of phenols interaction with this inflammation signaling system, since expression of some genes involved in NF-κB/MAPK/AP-1 signaling pathways was also modulated after phenol-rich olive oil based breakfast. NF-κB is a transcription factor activated by pro-inflammatory cytokines [34] and oxidative stress mediators [35]. Recently Pierce et al. have demonstrated that NF-κB activation is important in mediating vascular endothelial dysfunction in obese humans [36]. The product of *SGK1 *gene, encoding a serum/glucocorticoid regulated kinase with a role in stress response and by itself being a downstream target for PI_3_K signaling, enhances nuclear NF-κB activity by phosphorylating an inhibitory kinase IKKα [37]; so repression on expression of *SGK1 *gene by olive oil phenols would decrease the NF-κB activation. In addition, *NFKBIA *gene, which encodes to IκBα, a member of an inhibitory IκB family that retains NFκB into the cytoplasm, remained under-expressed after acute intake of phenol-rich olive oil. It has been reported that NF-κB binds to the IκBα promoter in order to activate its transcription [38]. Thus, this negative feedback mechanism results in rapid cycles of inhibition and stimulation of NF-κB where a decrease on NF-κB activation is accompanied by a reduction on *NFKBIA *gene expression, as observed in our results. The hypothesis that NF-κB activation is decreased by olive oil phenols is also supported by two *in vivo *studies which showed reduced NF-κB activation after olive oil consumption [8,9]. Additionally, *in vitro *studies showing attenuated NF-κB activation by resveratrol support the hypothesis that this pathway employs a shared mechanism by which polyphenols reduce expression of genes encoding inflammatory cytokines and adhesion molecules [39].

After intake of virgin olive oil with high content in phenolic compounds we found a decreased postprandial expression of *DUSP1 *and *DUSP2*. Those genes encode dual serine-threonine phosphatases, which down regulate members of p38, MAPK/ERK and SAPK/JNK, the three final effectors of the MAPK pathway [33]. In addition, *TRIB1*, another gene repressed by phenol-rich olive oil, is involved in MAPK signaling, participating in the activation of ERK proteins [40] and being up regulated in human atherosclerotic plaques [41]. Thus, reduction of *TRIB1 *expression by olive oil phenols could promote decreased ERK activation. This observation agrees with *in vitro *studies demonstrating that phenol compounds of green tea down regulate *PTGS2 *expression by decreasing ERK and p38 MAPK activation [42]. Our results allow us to hypothesize that olive oil phenols influence activation of AP-1, which consists of a variety of heterodimers of Jun, Fos and activating transcription factor families [43], by means of two different mechanisms: a) one direct, involving repression of *JUN*, *JUNB *and *FOSB *as observed after phenol-rich olive oil intake; and/or b) another one indirect, through MAPK pathway, where the relative intensity and duration of activation determine the type of response.

Lastly, biomedical literature and text mining tasks were performed to identify interactions of differentially expressed genes in PBMCs as response to phenol-rich VOO with conditions clustered around MetS such as obesity, hypertension, dyslipemia, hyperglycemia, or T2DM. Recently, Chen et al. have described a macrophage-enriched gene network (MEGN) of ~1237 genes referred as having causal relationship with complex-disease traits associated with MetS [44]. Thirteen genes share our set of differentially expressed genes in PBMCs after acute intake of phenol-rich olive oil and MEGN: *JUN*, *RGS1*, *CXCL2*, *ANXA3*, *RASGEF1B*, *CD83*, *CA2*, *EGR2*, *DIAPH3*, *CCL3*, and *TLR7*, *PSAP *and *IFIT3*. De Mello et al. assessed individuals with both impaired glucose metabolism and MetS on how long-term weight loss affects expression of cytokines in PBMCs. Weight reduction resulted in a decrease in of *IL1B *expression [45]. Kaiser et al. showed by microarrays analysis in PBMCs a set of 22 over-expressed genes in T1DM and T2DM compared to healthy subjects [46]. Interestingly, 8 of the identified 22 over-expressed genes in T2DM were repressed by olive oil phenols, according to our intervention study (*IL1B*, *EGR2*, *EGR3*, *PTGS2*, *FOSB*, *CXCL1*, *SGK*, and *TRIB1*). In addition, *PBEF1*, another gene involved in the pathogenesis of T2DM [47], was also found repressed after consumption of phenols-rich olive oil. Taken together, this finding could lead to potential therapeutic implications in T2DM.

## Conclusions

Our study shows that intake of breakfast based in virgin olive oil being rich in phenol compounds is able to repress expression of several pro-inflammatory genes *in vivo*, thereby switching activity of PBMCs to a less deleterious inflammatory profile. These results provide at least a partial molecular basis for risk reduction of cardiovascular disease observed in Mediterranean countries, where VOO represents a main source of dietary fat. Admittedly, other lifestyle factors are also likely to contribute to lowered risk of CVD in this region. Nonetheless, our data suggest that mechanisms by which these micronutrients in phenol-rich olive oil would exert their anti-inflammatory effect could involve pathways related to NF-κB/AP-1, cytokine-cytokine receptor interaction, arachidonic acid metabolism, and MAPK. These findings strengthen the relationship between inflammation, obesity and diet and provide evidence at transcription level of control of healthy effects derived from VOO consumption in humans. However, it would be interesting to evaluate whether these beneficial effects are maintained after prolonged feeding and if these effects are carried out by one or several olive oil phenolic compounds, or if they are consequence of a synergic effect of the total phenolic fraction.

## Methods

### Subjects

Twenty (56 years old; range, 40-70) subjects (9 men, 11 women) from the Lipids and Atherosclerosis Unit at Hospital Universitario Reina Sofía (Cordoba, Spain) participated in this study.

All subjects fulfilled three or more of the proposed criteria proposed by the Third Report of the National Cholesterol Education Program (NCEP) Expert Panel on Detection, Evaluation, and Treatment of High Blood Cholesterol in Adults (Adult Treatment Panel III) for MetS: a) central obesity (waist circumference > 102 cm in men or > 88 cm in women); b) high blood pressure ≥ 130/85 mmHg or documented use of antihypertensive therapy; c) high fasting glucose (≥ 100 mg.dL^-1^); d) hypertriglyceridemia (≥ 150 mg.dL^-1^), and e) low high density lipoprotein cholesterol (HDLc) (< 40 mg.dL^-1^ for males or 50 mg.dL^-1^ for females). Subjects had an average body mass index (BMI) of 38.98 (27.95-44.42) kg.m^-2^, waist perimeter of 132.2 cm (103.0-137.0), systolic blood pressure of 146 ± 19 mmHg, diastolic blood pressure of 89 ± 10 mmHg, plasma glucose levels of 102.8 ± 17.0 mg.dL^-1^, plasma insulin levels of 11.5 ± 6.5 mU.L^-1^, total cholesterol (TC) plasma levels of 201.7 ± 27.1 mg.dlL-1 triacylglyceride (TAG) plasma levels of 185 ± 78 mg.mL-1, low density lipoprotein cholesterol (LDLc) levels of 120.3 ± 19.7 mg.dL^-1^ and high density lipoprotein cholesterol (HDLc) levels of 50.7 ± 11.8 mg.dL-1. No subjects showed evidence of chronic diseases (hepatic, renal, thyroid, cardiac), smoking, alcohol consumption, or family history of cardiovascular disease of early onset, nor were they taking drugs. The Human Investigation Review Committee at Reina Sofia University Hospital approved this study. All participants gave informed consent before joining the study.

### Study design and dietary intervention

Before the first breakfast intervention, participants followed a 6-week washout period in which they were instructed to not take vitamins, soy supplements, or any drug. To eliminate the potential effect that might exist in their usual dietary habits, all subjects followed a low-fat, carbohydrate rich (CHO) diet during this period through the end of the study. Compliance with the CHO diet was assessed after two and four weeks using a three-day recording and a food frequency questionnaire. 24 hours prior to each breakfast intervention, participants were instructed to avoid consuming phenol-rich foods such as fruit or juices, wine, grape juice, chocolate, coffee, tea, olive oil, or soy, and to refrain from intense physical exercise. After a 12-h fast and following a randomized sequential crossover design with one-week washout period, participants reported to the hospital and received two fat meals consisting of 60 grams of white bread, 40 mL of VOO (CANOLIVA^®^, Antonio Cano e Hijos™, Cordoba, Spain) with high (398 ppm) or low (70 ppm) content in phenolic compounds, and 60,000 IU of vitamin A per m^2 ^of body surface. Throughout the 4-hour duration of the study session, subjects performed no physical activity, nor did they consume anything but water.

### Olive oil characteristics

Olive oil with low content in phenolic compounds was obtained by physical extraction of most phenolic compounds from the high-phenol olive oil, so that both oils retained a similar composition of the remaining macro- and micro-nutrients with the exception of phenolic content (70 ppm vs 398 ppm) (data not shown). Hydroxytyrosol content was 0.2 μmol.g^-1 ^and 45.4 μmol.g^-1^, respectively in low-phenol and high-phenol olive oil. High-phenol olive oil was washed in separating funnels with the same volume of distilled water. This operation was repeated seven times at room temperature under reduced light to avoid oxidation. Fatty acid composition of oils was determined by gas chromatography in a Perkin-Elmer Autosystem (SGE Scientific, Australia) according to EU Regulation 2568/91 (CE, 1991). Tocopherol composition was analyzed by HPLC in a Perkin-Elmer HPLC, applying IUPAC method 2432 (IUPAC, 1992).

### Sample collection

Venous blood samples were obtained at 0, 30, 60, 120 and 240 min after consumption of each breakfast. Samples from fasting and postprandial states were collected in tubes containing 1 g EDTA/L and stored in containers with iced water and kept in the dark. Special care was taken to avoid exposure to air, light, and ambient temperature. Plasma was separated from whole blood by low-speed centrifugation at 1500 × *g *for 15 min at 4°C within the hour after extraction.

### Lipid analysis and biochemical determinations

Lipid parameters were assessed with the modular autoanalyzer DDPPII Hitachi (Roche, Basel, Switzerland), using specific reagents (Boehringer-Mannheim, Mannheim, Germany). Determinations of TC and TAG levels were made by colorimetric enzymatic methods [48,49]; of HDLc by colorimetric assay [50]; of LDLc by the Friedewald formula based on CT, TAG, and HDLc values [51]. Plasma glucose concentrations were measured with a Hitachi 917 analyzer (Boehringer Mannheim, Mannheim, Germany) by the glucose oxidase method (GOD-PAP). Plasma insulin concentrations were measured by microparticle enzyme immunoassay (Abbott Diagnostics, Matsudo-shi, Japan). Nonsterified fatty acid concentrations were measured by enzymatic colorimetric assay (Roche Diagnostics, Penzberg, Germany).

### Isolation of PBMCs

PBMCs were isolated within 2 hours after blood draw from 30 mL EDTA anticoagulated blood samples taken 4 hours after consumption of each olive oil-based breakfast. Buffy coats were diluted 1:2 in PBS, and cells were separated in 5 mL Ficoll gradient (lymphocyte isolation solution, Rafer) by centrifugation at 2000 × *g *for 30 min. PBMCs were collected and washed twice with cold PBS. Harvested PBMCs were preserved in liquid nitrogen and stored at -80°C prior to RNA extraction.

### RNA extraction and microarray samples preparation

Total RNA was extracted from mononuclear cells with TRI Reagent (Sigma-Aldrich, Inc., St. Louis, MO, USA) and purified with RNeasy MiniElute Cleanup Kit (Qiagen, Hilden, Germany). Recovered RNA was quantified using a Nanodrop ND-1000 v3.5.2 spectrophotometer (Nanodrop Technology^®^, Cambridge, UK). RNA integrity was assessed using 1.6% agarose gel, 1× TBE. RNA was judged suitable for array hybridization only if samples exhibited intact bands corresponding to 18S and 28S ribosomal RNAs.

### Microarray analysis design

Gene expression profiles were generated using the *4x44K *glass slide *Whole Human Genome Oligo Microarray G4112A *(Agilent Technologies Inc., Santa Clara, CA, USA). Each microarray uses 45,220 probes to interrogate 30,886 unique human genes and transcripts. Two sets of dye-swapped experiments were performed to total four replicate hybridizations per subject (totals 80 microarray experiments). Each array compared total RNA from PBMCs obtained 4 hours after intake of virgin olive oil with high phenolic content with RNA obtained 4 hours after low-phenol virgin olive oil consumption.

### Synthesis and labeling of cDNA

RNA samples were labeled using the *SuperScript Indirect RNA Amplification System *(Invitrogen Inc., Carlsbad, CA, USA) according to the manufacturer's instructions. To avoid confounding by extraneous factors, all experiments were performed in a single batch and processed by one researcher on the same day for each step. Dye incorporation rates were assessed with a Nanodrop^® ^ND-1000 v3.5.2 spectrophotometer (Nanodrop Technology^®^, Cambridge, UK) and found to be between 8 and 12 pmol. μL^-1^.

### Microarray hybridation and scan protocol

Differentially labeled aRNA samples were cohybridized on microarray slides. Hybridization was performed using the *Gene Expression Hybridization Kit *(Agilent Technologies Inc., Santa Clara, CA, USA) following the manufacturer's instructions. Microarray images of each slide were obtained with a Gene Pix 4000B scanner (Axon Instruments, Union City, CA, USA). Image quantization was performed using Agilent Feature Extraction Software v9.5 (Agilent Technologies Inc., Santa Clara, CA, USA).

### Preprocessing and normalization of the microarray data

Background adjusted signals (BGSubSignal) were calculated by the Agilent Feature Extraction Software (v9.5) and filtered on the flag IsWellAboveBG. Log_2 _dye swapped (red/green) mean-centered signal ratio data were normalized within-array and between-array by Lowess and quantile methods respectively, both implemented in the R Limma package (GLP).

### Differential gene expression analysis

Identification of genes whose expression could be regulated by virgin olive oil phenols was done by comparing microarray results from PBMCs obtained at 4 hours after high and low phenol olive oil ingestion. Differential gene expression analysis was performed by fitting of gene-wise linear models to microarray data with R Limma package (GLP). For considering a gene as differentially expressed, filtering criteria were used combining M value (signal log_2 _ratio) greater than 0.4 (over-expressed) or lower than -0.4 (under-expressed) (by 1.32 and -1.32-fold changes, respectively), and statistical significance (*p*) of the fitted linear model of ≤ 0.01 for every gene. Results were adjusted by dye-swap effects and by False Discovery Rate (FDR) using Benjamini and Hochberg method.

### MIAME Gene Expression Omnibus (GEO) database

Raw microarray data have been curated and accepted in GEO (a public repository for microarray data, aimed at storing MIAME (Minimum Information About Microarray Experiments) compliant data in accordance with MGED (Microarray Gene Expression Data) recommendations. Access to data concerning this study may be found under GEO experiment accession number GSE15812 http://www.ncbi.nlm.nih.gov/geo/query/acc.cgi?token=pjihtumyoyogmhy&acc=GSE15812.

### qRT-PCR analysis of differentially expressed genes

PCR analyses were performed using Mx3005 Thermocycler (Agilent Technologies, La Jolla, CA) and *iQ SYBR*^®^*Green Supermix *(Bio-Rad Laboratories, Inc., Hercules, CA, USA) commercial kit in a final volume of 20 μl with 10 pmol of each primer. Each reaction was performed on 1 μl of 1:5 (v/v) dilution of the first cDNA strand, synthesized using 1 μg of total RNA by the commercial kit *iScript*^® ^*cDNA Synthesis Kit *(Bio-Rad Laboratories, Inc., Hercules, CA, USA) according to the manufacturer's instruction. The reaction was incubated at 96°C for 3 min, followed by 40 cycles of 30 s at 96°C, 30 s at 62°C, 20 s at 72°C and 10 s at 80°C where fluorescence was measured to avoid primer-dimer and background signals. Primers used were: ERG1-Up (5'-CTACGAGCACCTGACCGCAGAG-3'), ERG1-Low (5'-CCAGGGAAAAGCGGCCAGTATAG-3'), JUN-Up (5'-GACCTTCTATGACGATGCCCTCAA-3'), JUN-Low (5'-ACGTCGGGCGAGGTGAGGAGGTC-3'), IL1B-Up (5'-GGGACAGGATATGGAGCAACAAGT-3'), IL1B-Low (5'-GCTTATCATCTTTCAACACGCAGGA-3'), PTGS2-Up (5'-ATGAGTTATGTCTTGACATCCAGATCAC-3'), PTGS2-Low (5'-CAAACATCATGTTTGAGCCCTGG-3'), RPL13-Up (5'-CCTGGAGGAGAAGAGGAAAGAGA-3') and RPL13-Low (5'-TTGAGGACCTCTGTGTATTTGTCAA-3'). Specificity of PCR amplifications was verified by a melting curve program (60-95°C with a heating rate of 0.5°C/s and a continuous fluorescence measurement) and analyzed by electrophoresis on a 1.6% agarose gel, TBE 1×. Primer efficiencies were: EGR1, 98%; PTGS2, 101%; IL1B, 96%; JUN, 108%; RPL13a, 95%. Expression values were obtained as relative expression of the target gene versus the constitutively expressed RPL13a gene (relative expression = 2^-(Ct, Target gene-Ct, Reference gene)^).

### Statistical analysis

Several variables were transformed to define the metabolic postprandial response after acute intake of both types of olive oil. Incremental area under plasma concentration curve (iAUC) was calculated for all measured metabolic variables by the trapezoidal method. The normality of data was assessed using the Saphiro-Wilk test. Log transformation of data was performed when those variables were not normally distributed. Differences between iAUC after intake of high-phenol and low-phenol olive oils were analyzed using Wilcoxon-paired test.

For data analysis, gene expression data were presented in M-values [log_2_(ratio)] expressing a fold change after high-phenol olive oil intake compared to low-phenol olive oil consumption. The corresponding *p*-value, B-value, and B-probability are shown (Additional File 1). The data input for correlation analysis using Spearman's test were the log_2_(ratio) value for microarrays (M-value) and qRT-PCR in subject individuals.

All data presented in the text and tables are expressed as mean ± SD unless otherwise specified. Significance levels were set to *p*-values less than 0.01 for microarray data and less than 0.05 for all other analyses. All statistical tests were performed using R programming and statistical environment software (GLP).

## Abbreviations used

ppm: part per million; MetS: metabolic syndrome; CVD: cardiovascular disease; VOO: virgin olive oil; MD: Mediterranean Diet; PBMC: peripheral blood; mononuclear cells; MEGN: macrophage-enriched gene network.

## Authors' contributions

AC and JR contributed equally to this work. AC, JR, JL-M, and FP-J: designed research; AC, JMF, and MU: provision of study materials and subjects; AC, JR, JMF, AJ, and MS-G: performed research; LDP and MU: contributed new reagents/analytic tools; AC, AJ, and MS-G: collection and assembly of data; AC, JR, JMF, LDP, CM, PP-M, JL-M, and FP-J: analyzed data; CM, PP-M, JL-M, and FP-J: provided statistical expertise; AC: wrote the paper; JR, LDP, MU, JL-M and FP-J: critical review of the manuscript; FP-J: obtained funding. None of the authors had any personal or financial conflicts of interest.

## Supplementary Material

Additional file 1 **Description of differentially expressed genes in microarray analysis**. List of the differentially expressed genes identified by microarray analysis when comparing the intake of phenol-rich olive oil with low-phenol olive oil in mononuclear cells in patients with metabolic syndrome. Click here for file

Additional file 2 **Description of differentially expressed genes in gender analysis**. List of the differentially expressed genes when comparing the intake of phenol-rich olive oil with low-phenol olive oil in mononuclear cells in patients with metabolic syndrome in gender analysis. Click here for file
