# Supplementary material for: Gene expression changes in mononuclear cells in patients with metabolic syndrome after acute intake of phenol-rich virgin olive oil
Source: BMC Genomics. 2010 Apr 20;11:253. doi: 10.1186/1471-2164-11-253 (PMC2874810; doi:10.1186/1471-2164-11-253)
Supplement: Additional file 1 — Description of differentially expressed genes in microarray analysis. List of the differentially expressed genes identified by microarray analysis when comparing the intake of phenol-rich olive oil with low-phenol olive oil in mononuclear cells in patients with metabolic syndrome. [file 1471-2164-11-253-S1.DOC]

**Additional File 1. Description of differentially expressed genes in microarray analysis.**

| ***Overexpressed genes after intake of olive oil phenols*** | | |  |  |  |  |
| --- | --- | --- | --- | --- | --- | --- |
| ***Gene*** | ***GenBankID*** | ***Description*** | ***M-value**** | ***p-value*** | ***B-value*** | ***B-probability*** |
| ***Cytokine receptor*** | |  |  |  |  |  |
| CCR2 | NM_000647 | chemokine (C-C motif) receptor 2 | 0.416 | 0.00313055 | 0.476 | 61.69 |
| ***Transporter*** |  |  |  |  |  |  |
| SLC6A12 | NM_003044 | solute carrier family 6, member 12 | 0.408 | 0.00001598 | 8.226 | 99.97 |
| SLC4A1 (W) | NM_000342 | solute carrier family 4, anion exchanger, member 1 | 0.409 | 0.00015070 | 4.859 | 99.23 |
| ABCC13 | NR_003087 | ATP-binding cassette, sub-family C, member 13 | 0.443 | 0.00247545 | 0.797 | 68.92 |
| ***Membrane receptors*** | |  |  |  |  |  |
| TLR7 | NM_016562 | toll-like receptor 7 | 0.407 | 0.00019344 | 4.534 | 98.94 |
| GRIA4 | NM_000829 | glutamate receptor, ionotrophic, AMPA 4 | 0.434 | 0.00074170 | 2.596 | 93.06 |
| ***Membrane proteins*** | |  |  |  |  |  |
| PSAP (M) | NM_002778 | prosaposin | 0.437 | 0.00229454 | 0.905 | 71.20 |
| GYPB (W) | NM_002100 | glycophorin B | 0.602 | 0.00070669 | 2.670 | 93.52 |
| ***Enzymes*** |  |  |  |  |  |  |
| ALDH1A1 (M) | NM_000689 | aldehyde dehydrogenase 1 family, member A1 | 0.418 | 0.00117732 | 1.878 | 86.73 |
| CA1 (W) | NM_001738 | carbonic anhydrase I | 0.653 | 0.00022733 | 4.314 | 98.68 |
| ***Kinase*** |  |  |  |  |  |  |
| PDK4 (M) | NM_002612 | pyruvate dehydrogenase kinase, isozyme 4 | 0.452 | 0.00201227 | 1.103 | 75.08 |
| ***Peptidase*** |  |  |  |  |  |  |
| CPVL | NM_031311 | carboxypeptidase, vitellogenic-like | 0.455 | 0.00729726 | -0.703 | 33.11 |
| ***Metal ion binding*** | |  |  |  |  |  |
| SELENBP1(W) | NM_003944 | selenium binding protein 1 | 0.551 | 0.00007747 | 5.841 | 99.71 |
| ***Protein binding*** | |  |  |  |  |  |
| CSTA (M) | NM_005213 | cystatin A (stefin A) | 0.434 | 0.00039829 | 3.505 | 97.08 |
| FN1 | NM_212482 | fibronectin 1 | 0.553 | 0.00856086 | -0.838 | 30.19 |
| ***Signal transduction*** | |  |  |  |  |  |
| RAP1GAP | NM_002885 | RAP1 GTPase activating protein | 0.610 | 0.00385783 | 0.237 | 55.89 |
| ***Transcription regulators*** | |  |  |  |  |  |
| ANKRD22 (M) | NM_144590 | ankyrin repeat domain 22 | 0.517 | 0.00005076 | 6.546 | 99.86 |
| ***Hypothetical genes or non-annotated function genes*** | | |  |  |  |  |
| C8orf13  (FAM167A) | NM_053279 | chromosome 8 open reading frame 13 | 0.443 | 0.00582300 | -0.389 | 40.39 |
| IFIT3 | NM_001549 | interferon-induced protein | 0.468 | 0.00006371 | 6.159 | 99.79 |
|  | | |  |  |  |  |
| ***Underexpressed genes after intake of olive oil phenols*** | | |  |  |  |  |
| ***Gene*** | ***GenBankID*** | ***Description*** | ***M-value*** | ***p-value*** | ***B-value*** | ***B-probability*** |
| ***Cytokines*** |  |  |  |  |  |  |
| IL1B (M) | NM_000576 | interleukin 1, beta | -1.081 | 0.00187965 | 1.197 | 76.80 |
| PBEF1 (X)  (NAMPT) | NM_005746 | pre-B-cell colony enhancing factor 1 | -0.785 | 0.00022793 | 4.304 | 98.67 |
| IL6 (M) | NM_000600 | interleukin 6 | -0.621 | 0.00031279 | 3.855 | 97.93 |
| OSM (M) | NM_020530 | oncostatin M | -0.463 | 0.00104983 | 2.060 | 88.70 |
| ***Growth factors*** | |  |  |  |  |  |
| EREG (W) | NM_001432 | epiregulin | -0.773 | 0.00591099 | -0.417 | 39.71 |
| AREG | NM_001657 | amphiregulin | -0.578 | 0.00589778 | -0.413 | 39.81 |
| ***Cytokine ligand*** | |  |  |  |  |  |
| CXCL1 (W) | NM_001511 | chemokine (C-X-C motif) ligand 1 | -0.987 | 0.00099775 | 2.138 | 89.45 |
| CCL3L3 | NM_001001437 | chemokine (C-C motif) ligand 3-like 3 | -0.637 | 0.00457507 | -0.045 | 48.87 |
| CCL3 | NM_002983 | chemokine (C-C motif) ligand 3 | -0.622 | 0.00591099 | -0.417 | 39.73 |
| CXCL2 (X) | NM_002089 | chemokine (C-X-C motif) ligand 2 | -0.604 | 0.00015612 | 4.802 | 99.19 |
| CXCL3 (X) | NM_002090 | chemokine (C-X-C motif) ligand 3 | -0.559 | 0.00174509 | 1.295 | 78.50 |
| ***Cytokine receptor*** | |  |  |  |  |  |
| CXCR4 (X) | NM_001008540 | chemokine (C-X-C motif) receptor 4 | -0.611 | 0.00004847 | 6.632 | 99.87 |
| ***Transporter*** |  |  |  |  |  |  |
| SLC16A3 (M) | AF318321 | pp10472 mRNA, complete cds | -0.485 | 0.00171066 | 1.323 | 78.96 |
| ***Membrane receptor*** | |  |  |  |  |  |
| CD69 (M) | NM_001781 | CD69 molecule | -0.729 | 0.00065899 | 2.767 | 94.08 |
| TAS2R50 (X) | NM_176890 | taste receptor, type 2, member 50 | -0.447 | 0.00000051 | 12.859 | 100.0 |
| ***Ion channel*** |  |  |  |  |  |  |
| KCNV2 (W) | NM_133497 | potassium channel, subfamily V, member 2 | -0.416 | 0.00000331 | 10.418 | 100.0 |
| ***G-protein coupled receptor*** | |  |  |  |  |  |
| RGS1 (M) | NM_002922 | regulator of G-protein signalling 1 | -0.813 | 0.00318194 | 0.454 | 61.15 |
| ***Membrane proteins*** | |  |  |  |  |  |
| CD83 (M) | NM_004233 | CD83 molecule | -0.576 | 0.00161103 | 1.414 | 80.43 |
| CNTNAP3 | NM_033655 | contactin associated protein-like 3 | -0.532 | 0.00669077 | -0.561 | 36.33 |
| LMOD1 (X) | NM_012134 | leiomodin 1 | -0.527 | 0.00000011 | 14.941 | 100.0 |
| TMEM88 (X) | NM_203411 | transmembrane protein 88 | -0.485 | 0.00041487 | 3.445 | 96.91 |
| ***Enzymes*** |  |  |  |  |  |  |
| PTGS2 (W) | NM_000963 | prostaglandin-endoperoxide synthase 2 | -0.967 | 0.00148104 | 1.537 | 82.31 |
| SOD2 (X) | BC016934 | superoxide dismutase 2, mitochondrial | -0.776 | 0.00116864 | 1.889 | 86.87 |
| KRT23 (W) | NM_015515 | keratin 23 (histone deacetylase inducible) | -0.512 | 0.00355213 | 0.296 | 57.35 |
| THEM5 (W) | NM_182578 | thioesterase superfamily member 5 | -0.439 | 0.00002036 | 7.917 | 99.96 |
| CA2 (W) | NM_000067 | carbonic anhydrase II | -0.414 | 0.00000157 | 11.458 | 100.0 |
| PLA2G1B (X) | NM_000928 | phospholipase A2, group IB | -0.412 | 0.00000222 | 11.041 | 100.0 |
| ANXA3 (W) | NM_005139 | annexin A3 (phospholipase A2 inhibitor) | -0.404 | 0.00006864 | 6.039 | 99.76 |
| CYCS | NM_018947 | cytochrome c, somatic | -0.448 | 0.00202134 | 1.094 | 74.92 |
| ***Phosphatases*** | |  |  |  |  |  |
| DUSP1 (W) | NM_004417 | dual specificity phosphatase 1 | -0.708 | 0.00475031 | -0.103 | 47.44 |
| PHACTR1 (M) | AB051520 | mRNA for KIAA1733 protein | -0.594 | 0.00201902 | 1.099 | 75.01 |
| DUSP2 (X) | NM_004418 | dual specificity phosphatase 2 | -0.573 | 0.00005556 | 6.385 | 99.83 |
| PPP1R15A (X) | NM_014330 | protein phosphatase 1, inhibitory subunit 15A | -0.519 | 0.00024146 | 4.217 | 98.55 |
| ***Kinases*** |  |  |  |  |  |  |
| SGK1 | NM_005627 | serum/glucocorticoid regulated kinase | -0.609 | 0.00038645 | 3.550 | 97.21 |
| TRIB1 (W) | NM_025195 | tribbles homolog 1 | -0.456 | 0.00326870 | 0.410 | 60.12 |
| ***Metal ion binding*** | |  |  |  |  |  |
| PVALB (X) | NM_002854 | parvalbumin | -0.494 | 0.00000000 | 22.989 | 100.0 |
| LIMS3 (X) | NM_033514 | LIM and senescent cell antigen-like domains 3 | -0.460 | 0.00000225 | 10.929 | 100.0 |
| TNNC2 (X) | NM_003279 | troponin C type 2 | -0.428 | 0.00002392 | 7.697 | 99.95 |
| ***Protein binding*** | |  |  |  |  |  |
| PER1 (M) | BC028207 | period homolog 1 | -0.625 | 0.00336160 | 0.376 | 59.30 |
| DIAPH3 | NM_030932 | diaphanous homolog 3 | -0.532 | 0.00209396 | 1.111 | 75.24 |
| DFNB31 | AK056190 | cDNA FLJ31628 fis, clone NT2RI2003344 | -0.528 | 0.00074170 | 2.604 | 93.11 |
| NPHS2 (w) | NM_014625 | nephrosis 2, idiopathic, steroid-resistant (podocin) | -0.522 | 0.00000943 | 8.834 | 99.99 |
| APR-2 (X)  (C17orf88) | BC069097 | apoptosis related protein | -0.453 | 0.00000022 | 14.013 | 100.0 |
| ***Signal transduction*** | |  |  |  |  |  |
| RASGEF1B | BX648337 | cDNA DKFZp686K235 | -0.990 | 0.00144119 | 1.580 | 82.92 |
| SDCBP (X) | AK128645 | cDNA FLJ46804 fis, clone TRACH3032570 | -0.449 | 0.00353463 | 0.302 | 57.48 |
| TAGAP (X) | NM_138810 | T-cell activation GTPase activating protein | -0.403 | 0.00019345 | 4.533 | 98.94 |
| ***Signaling*** |  |  |  |  |  |  |
| TNFAIP3 | NM_006290 | tumor necrosis factor, alpha-induced protein 3 | -0.644 | 0.00199499 | 1.114 | 75.28 |
| NFKBIA | NM_020529 | nuclear factor of kappa light polypeptide gene enhancer in B-cells inhibitor, alpha | -0.629 | 0.00286694 | 0.594 | 64.42 |
| SOCS3 (X) | NM_003955 | suppressor of cytokine signaling 3 | -0.589 | 0.00010010 | 5.485 | 99.59 |
| NFKBIZ (X) | NM_031419 | nuclear factor of kappa light polypeptide gene enhancer in B-cells inhibitor, zeta | -0.508 | 0.00059701 | 2.911 | 94.84 |
| ***Cell cycle regulator*** | |  |  |  |  |  |
| G0S2 (M) | NM_015714 | G0/G1switch 2 | -1.455 | 0.00130094 | 1.732 | 84.96 |
| CDKN2A (X) | NM_058197 | cyclin-dependent kinase inhibitor 2A | -0.411 | 0.00000008 | 15.787 | 100.0 |
| ***Ligand-dependent nuclear receptors*** | | |  |  |  |  |
| NR4A2 (W) | NM_006186 | nuclear receptor subfamily 4, group A, member 2 | -1.078 | 0.00111640 | 1.965 | 87.70 |
| NR4A1 (M) | NM_002135 | nuclear receptor subfamily 4, group A, member 1 | -0.580 | 0.00732569 | -0.709 | 32.98 |
| ***Transcription regulators*** | |  |  |  |  |  |
| EGR2 (W) | NM_000399 | early growth response 2 | -1.434 | 0.00091481 | 2.266 | 90.61 |
| EGR1 (X) | NM_001964 | early growth response 1 | -1.393 | 0.00035479 | 3.665 | 97.50 |
| FOSB (W) | NM_006732 | FBJ murine osteosarcoma viral oncogene homolog B | -1.309 | 0.00148294 | 1.535 | 82.28 |
| EGR3 (W) | NM_004430 | early growth response 3 | -1.062 | 0.00084380 | 2.425 | 91.87 |
| JUN (X) | NM_002228 | v-jun sarcoma virus 17 oncogene homolog | -0.823 | 0.00052106 | 3.106 | 95.71 |
| JUNB (X) | NM_002229 | jun B proto-oncogene | -0.523 | 0.00032703 | 3.791 | 97.79 |
| TRIM29 (X) | NM_012101 | tripartite motif-containing 29 | -0.520 | 0.00000009 | 15.576 | 100.0 |
| KLF6 (X) | NM_001008490 | Kruppel-like factor 6 | -0.483 | 0.00507374 | -0.198 | 45.06 |
| ZFP36 (X) | NM_003407 | zinc finger protein 36, C3H type | -0.466 | 0.00076127 | 2.560 | 92.83 |
| MXD1 (W) | NM_002357 | MAX dimerization protein 1 | -0.454 | 0.00010098 | 5.474 | 99.58 |
| MLXIPL (X) | NM_032954 | MLX interacting protein-like | -0.438 | 0.00000077 | 12.299 | 100.0 |
| HOXB13 (X) | NM_006361 | homeobox B13 | -0.425 | 0.00000011 | 15.104 | 100.0 |
| ***Hypothetical genes*** ***or non-annotated function genes*** | | |  |  |  |  |
| LOC284454 (X) | AL832183 | cDNA DKFZp686D0720 | -0.702 | 0.00190451 | 1.181 | 76.52 |
| FLJ22659 (W) | AK026312 | cDNA: FLJ22659 fis, clone HSI07953 | -0.590 | 0.00360587 | 0.279 | 56.92 |
| LOC389607 (W) | NM_001013651 | hypothetical gene supported by AK128318 | -0.552 | 0.00000962 | 8.808 | 99.99 |
| IER2 (X) | NM_004907 | immediate early response 2 | -0.543 | 0.00007085 | 5.990 | 99.75 |
| AY029066 (W) | AY029066 | Humanin (HN1), likely mitochondrial 16S rRNA | -0.540 | 0.00008548 | 5.705 | 99.67 |
| IER3 | NM_003897 | immediate early response 3 | -0.535 | 0.00133114 | 1.690 | 84.43 |
| LRRC41 (X) | AK024051 | cDNA FLJ13989 fis, clone Y79AA1002083 | -0.508 | 0.00000264 | 10.684 | 100 |
| AF351612  (NSUN3) | AF351612 | UG0651E06 mRNA, complete cds | -0.497 | 0.00233700 | 0.878 | 70.63 |
| C19orf19 (X)  (ODF3L2) | NM_182577 | chromosome 19 open reading frame 19 | -0.460 | 0.00000247 | 10.776 | 100 |
| ATXN7L2 (X) | NM_153340 | ataxin 7-like 2 | -0.457 | 0.00000029 | 13.608 | 100 |
| KIAA1922 (W)  (CROCCL2) | BC033082 | KIAA1922 protein, mRNA | -0.455 | 0.00000138 | 11.639 | 100 |
| C10orf63 (X)  (ENKUR) | NM_145010 | chromosome 10 open reading frame 63 | -0.452 | 0.00000346 | 10.360 | 100 |
| C21orf7 (W) | NM_020152 | chromosome 21 open reading frame 7 | -0.425 | 0.00046761 | 3.263 | 96.31 |

M-values [log2(ratio)] expressing a fold change after high-phenol olive oil acute intake compared to low-phenol olive oil consumption. The table shows the differentially expressed genes in the global analysis. It is noted the genes showing significant changes for gender analysis: **M**: differentially expressed in the males analysis; **W**: differentially expressed in the females analysis; **X**: differentially expressed in the men and women analysis.
