# Supplementary material for: Gene expression changes in mononuclear cells in patients with metabolic syndrome after acute intake of phenol-rich virgin olive oil
Source: BMC Genomics. 2010 Apr 20;11:253. doi: 10.1186/1471-2164-11-253 (PMC2874810; doi:10.1186/1471-2164-11-253)
Supplement: Additional file 2 — Description of differentially expressed genes in gender analysis. List of the differentially expressed genes when comparing the intake of phenol-rich olive oil with low-phenol olive oil in mononuclear cells in patients with metabolic syndrome in gender analysis. [file 1471-2164-11-253-S2.DOC]

**Additional File 2. Description of differentially expressed genes in gender analysis**.

| ***Overexpressed genes after intake of olive oil phenols in men*** | | | | |  |  |
| --- | --- | --- | --- | --- | --- | --- |
| ***Gene*** | ***GenBankID*** | ***Description*** | ***M-value*** | ***p-value*** | ***B-value*** | ***B-probability*** |
| ***Cytokines*** | |  |  |  |  |  |
| TNFSF13B | NM_006573 | tumor necrosis factor (ligand) superfamily, member 13b | 0.435 | 0.00904142 | 0.425 | 29.82 |
| TNFSF13 | NM_172088 | tumor necrosis factor (ligand) superfamily, member 13 | 0.721 | 0.00984741 | 0.238 | 19.22 |
| ***Growth factor*** | |  |  |  |  |  |
| VEGF | NM_001025366 | vascular endothelial growth factor | 0.479 | 0.00584938 | 1.375 | 57.89 |
| ***Cytokine ligand*** | |  |  |  |  |  |
| CXCL10 | NM_001565 | chemokine (C-X-C motif) ligand 10 | 0.793 | 0.00236775 | 3.616 | 78.34 |
| CXCL9 | NM_002416 | chemokine (C-X-C motif) ligand 9 | 0.454 | 0.00818969 | 0.643 | 39.14 |
| ***Transporter*** | |  |  |  |  |  |
| TM9SF2 | NM_004800 | transmembrane 9 superfamily member 2 | 0.405 | 0.00771025 | 0.792 | 44.20 |
| SLC43A3 | NM_199329 | solute carrier family 43, member 3 | 0.416 | 0.00598379 | 1.314 | 56.78 |
| AP1S2 | NM_003916 | adaptor-related protein complex 1, sigma 2 subunit | 0.633 | 0.00705724 | 0.977 | 49.42 |
| ***Transmembrane receptor*** | |  |  |  |  |  |
| HLA-DRA | NM_019111 | major histocompatibility complex, class II, DR alpha | 0.425 | 0.00485636 | 1.861 | 65.05 |
| CD300C | NM_006678 | CD300c molecule | 0.455 | 0.0077175 | 0.781 | 43.85 |
| HLA-DRB3 | BC106057 | major histocompatibility complex, class II, DR beta 3 | 0.571 | 0.00965619 | 0.274 | 21.51 |
| FCGR3B | NM_000570 | Fc fragment of IgG, low affinity IIIb, receptor | 0.666 | 0.00702281 | 0.992 | 49.80 |
| ***Membrane proteins*** | |  |  |  |  |  |
| ERMAP | NM_001017922 | erythroblast membrane-associated protein | 0.403 | 0.00950836 | 0.319 | 24.18 |
| CDC42SE1 | NM_020239 | CDC42 small effector 1 | 0.412 | 0.00698219 | 1.013 | 50.32 |
| CD33 | NM_001772 | CD33 molecule | 0.444 | 0.00636676 | 1.179 | 54.11 |
| PLXNC1 | AB208934 | mRNA for plexin C1 variant protein | 0.444 | 0.00423597 | 2.152 | 68.27 |
| EPB41L3 | NM_012307 | erythrocyte membrane protein band 4.1-like 3 | 0.454 | 0.00535516 | 1.596 | 61.48 |
| GPM6B | NM_001001996 | glycoprotein M6B | 0.461 | 0.0016385 | 5.476 | 84.56 |
| CD1D | NM_001766 | CD1d molecule | 0.466 | 0.00275731 | 3.135 | 75.82 |
| LILRA4 | NM_012276 | leukocyte immunoglobulin-like receptor, subfamily A (with TM domain), member 4 | 0.492 | 0.00967662 | 0.268 | 21.14 |
| LILRA6 | NM_024318 | leukocyte immunoglobulin-like receptor, subfamily A (with TM domain), member 6 | 0.575 | 0.00699947 | 1.008 | 50.20 |
| LILRA2 | NM_006866 | leukocyte immunoglobulin-like receptor, subfamily A (with TM domain), member 2 | 0.605 | 0.00729349 | 0.915 | 47.78 |
| PSAP | NM_002778 | prosaposin | 0.731 | 0.00423597 | 2.153 | 68.28 |
| ***Enzymes*** | |  |  |  |  |  |
| ALDH1A1 | NM_000689 | aldehyde dehydrogenase 1 family, member A1 | 0.778 | 0.0052962 | 1.62 | 61.83 |
| RHOQ | NM_012249 | ras homolog gene family, member Q | 0.404 | 0.00580878 | 1.395 | 58.25 |
| ACAD10 | NM_025247 | acyl-Coenzyme A dehydrogenase family, member 10 | 0.405 | 0.00766355 | 0.807 | 44.66 |
| NDUFS2 | NM_004550 | NADH dehydrogenase | 0.406 | 0.00598379 | 1.314 | 56.78 |
| PLSCR1 | NM_021105 | phospholipid scramblase 1 | 0.407 | 0.00830821 | 0.608 | 37.81 |
| CTAGE5 | NM_203355 | CTAGE family, member 5 | 0.428 | 0.00494384 | 1.794 | 64.21 |
| SUV420H1 | NM_016028 | suppressor of variegation 4-20 homolog 1 (Drosophila) | 0.429 | 0.0025857 | 3.312 | 76.81 |
| RAB1A | NM_004161 | RAB1A, member RAS oncogene family | 0.436 | 0.00191727 | 4.172 | 80.67 |
| GCH1 | NM_000161 | GTP cyclohydrolase 1 | 0.455 | 0.00225853 | 3.681 | 78.64 |
| GBP1 | NM_002053 | guanylate binding protein 1 | 0.471 | 0.0016385 | 5.498 | 84.61 |
| WARS | NM_004184 | tryptophanyl-tRNA synthetase | 0.477 | 0.00191727 | 4.165 | 80.64 |
| KYNU | NM_003937 | kynureninase (L-kynurenine hydrolase) | 0.480 | 0.00282583 | 3.066 | 75.41 |
| ASAH1 | NM_004315 | N-acylsphingosine amidohydrolase | 0.484 | 0.00256245 | 3.344 | 76.98 |
| LIG4 | NM_002312 | ligase IV, DNA, ATP-dependent | 0.578 | 0.00881657 | 0.472 | 32.07 |
| OAS1 | NM_002534 | 2',5'-oligoadenylate synthetase 1 | 0.581 | 0.00819418 | 0.64 | 39.02 |
| ***Phosphatase*** | |  |  |  |  |  |
| INPP1 | NM_002194 | inositol polyphosphate-1-phosphatase | 0.466 | 0.00879592 | 0.488 | 32.80 |
| ***Kinases*** | |  |  |  |  |  |
| UHMK1 | NM_175866 | U2AF homology motif (UHM) kinase 1 | 0.430 | 0.00410008 | 2.23 | 69.04 |
| PDK4 | NM_002612 | pyruvate dehydrogenase kinase, isozyme 4 | 1.171 | 0.0035035 | 2.503 | 71.45 |
| PAK1 | NM_002576 | p21/Cdc42/Rac1-activated kinase 1 | 0.405 | 0.0050706 | 1.743 | 63.54 |
| NAGK | NM_017567 | N-acetylglucosamine kinase | 0.463 | 0.00580878 | 1.397 | 58.28 |
| TK2 | NM_004614 | thymidine kinase 2, mitochondrial | 0.468 | 0.00513094 | 1.706 | 63.05 |
| FGR | NM_005248 | Gardner-Rasheed feline sarcoma viral (v-fgr) oncogene homolog | 0.511 | 0.00236722 | 3.625 | 78.38 |
| HCK | NM_002110 | hemopoietic cell kinase | 0.648 | 0.00771048 | 0.784 | 43.95 |
| ***Peptidases*** | |  |  |  |  |  |
| CTSL | NM_001912 | cathepsin L | 0.418 | 0.00696162 | 1.019 | 50.47 |
| LAP3 | NM_015907 | leucine aminopeptidase 3 | 0.440 | 0.00178952 | 4.688 | 82.42 |
| PSMB9 | NM_002800 | proteasome (prosome, macropain) subunit, beta type, 9 | 0.455 | 0.00975101 | 0.257 | 20.45 |
| SCPEP1 | NM_021626 | serine carboxypeptidase 1 | 0.471 | 0.00600278 | 1.309 | 56.69 |
| PSEN1 | NM_000021 | presenilin 1 | 0.485 | 0.00607376 | 1.278 | 56.10 |
| FGL2 | NM_006682 | fibrinogen-like 2 | 0.707 | 0.0056947 | 1.454 | 59.25 |
| CTSS | NM_004079 | cathepsin S | 0.721 | 0.00443499 | 2.062 | 67.34 |
| ***Protein Binding*** | |  |  |  |  |  |
| CSTA | NM_005213 | cystatin A (stefin A) | 0.734 | 0.00702281 | 0.991 | 49.77 |
| ***Cell cycle*** | |  |  |  |  |  |
| GPIAP1 | NM_005898 | GPI-anchored membrane protein 1 | 0.421 | 0.00537176 | 1.586 | 61.33 |
| ***Translation Regulator*** | |  |  |  |  |  |
| EEF1A1 | NM_001402 | eukaryotic translation elongation factor 1 alpha 1 | 0.576 | 0.00580878 | 1.403 | 58.39 |
| ***Transcription Regulator*** | |  |  |  |  |  |
| BLZF1 | NM_003666 | basic leucine zipper nuclear factor 1 | 0.419 | 0.00143042 | 6.188 | 86.09 |
| LCORL | AK055258 | cDNA FLJ30696 fis | 0.430 | 0.00449422 | 2.022 | 66.91 |
| CEBPB | NM_005194 | CCAAT/enhancer binding protein (C/EBP), beta (CEBPB) | 0.472 | 0.00881039 | 0.484 | 32.61 |
| SAMD4A | AB028976 | mRNA for KIAA1053 protein | 0.508 | 0.00734961 | 0.89 | 47.09 |
| HHEX | NM_002729 | homeobox, hematopoietically expressed | 0.605 | 0.00992531 | 0.217 | 17.83 |
| STAT1 | NM_007315 | signal transducer and activator of transcription 1 | 0.691 | 0.00310386 | 2.808 | 73.74 |
| ANKRD22 | NM_144590 | ankyrin repeat domain 22 | 0.771 | 0.00143042 | 6.133 | 85.98 |
| ***Hypothetical genes or non-annotated function genes*** | | |  |  |  |  |
| YTHDF3 | NM_152758 | YTH domain family, member 3 | 0.440 | 0.00979266 | 0.251 | 20.06 |
| RPL15 | NM_002948 | ribosomal protein L15 | 0.402 | 0.00586449 | 1.367 | 57.75 |
| FER1L3 | NM_013451 | fer-1-like 3, myoferlin (C. elegans) | 0.405 | 0.00191727 | 4.139 | 80.54 |
| C9orf72 | NM_145005 | chromosome 9 open reading frame 72 | 0.408 | 0.00975101 | 0.257 | 20.45 |
| CKAP2 | NM_018204 | cytoskeleton associated protein 2 | 0.409 | 0.00825736 | 0.621 | 38.31 |
| PSCD4 | NM_013385 | pleckstrin homology, Sec7 and coiled-coil domains 4 | 0.410 | 0.00732922 | 0.9 | 47.37 |
| PARP14 | NM_017554 | poly (ADP-ribose) polymerase family, member 14 | 0.415 | 0.00143042 | 6.157 | 86.03 |
| C8orf38 | NM_152416 | chromosome 8 open reading frame 38 | 0.416 | 0.00523222 | 1.659 | 62.39 |
| COP1 | NM_001017534 | caspase-1 dominant-negative inhibitor pseudo-ICE | 0.423 | 0.00412099 | 2.219 | 68.93 |
| MRC1L1 | NM_001009567 | mannose receptor, C type 1-like 1 | 0.424 | 0.00735848 | 0.883 | 46.89 |
| AOF1 | NM_153042 | amine oxidase (flavin containing) domain 1 | 0.428 | 0.00338907 | 2.582 | 72.08 |
| TRIM44 | NM_017583 | tripartite motif-containing 44 | 0.437 | 0.00178952 | 5.008 | 83.36 |
| PB1 | NM_018313 | polybromo 1 | 0.439 | 0.00539836 | 1.568 | 61.06 |
| C20orf106 | AK097528 | cDNA FLJ40209 fis, clone TESTI2020999 | 0.441 | 0.00941209 | 0.348 | 25.82 |
| HNRPA1 | NM_002136 | heterogeneous nuclear ribonucleoprotein A1 | 0.448 | 0.0050706 | 1.751 | 63.65 |
| C1orf63 | AK027318 | cDNA FLJ14412 fis, clone HEMBA1004669 | 0.459 | 0.00143042 | 6.395 | 86.48 |
| TM2D3 | NM_078474 | TM2 domain containing 3 (TM2D3), transcript variant 1 | 0.471 | 0.00293365 | 2.945 | 74.65 |
| BATF2 | NM_138456 | basic leucine zipper transcription factor, ATF-like 2 | 0.484 | 0.00511139 | 1.713 | 63.14 |
| C14orf108 | NM_018229 | chromosome 14 open reading frame 108 | 0.505 | 0.00881657 | 0.476 | 32.25 |
| FLJ11151 | NM_018340 | hypothetical protein | 0.508 | 0.00584938 | 1.375 | 57.89 |
| RP11-262H14.4 | NM_203448 | hypothetical protein MGC21881 | 0.520 | 0.00700539 | 0.998 | 49.95 |
| ZNF121 | NM_001008727 | zinc finger protein 121 | 0.524 | 0.00191727 | 4.141 | 80.55 |
| MS4A7 | NM_021201 | membrane-spanning 4-domains, subfamily A, member 7 | 0.555 | 0.00636215 | 1.182 | 54.17 |
| LILRP2 | NR_003061 | leukocyte immunoglobulin-like receptor pseudogene 2 | 0.557 | 0.00864832 | 0.523 | 34.34 |
| MOSPD2 | NM_152581 | motile sperm domain containing 2 | 0.563 | 0.00449672 | 2.013 | 66.81 |
| RBM7 | NM_016090 | RNA binding motif protein 7 | 0.570 | 0.00262979 | 3.261 | 76.53 |
| CLEC12A | NM_138337 | C-type lectin domain family 12, member A | 0.624 | 0.00757169 | 0.835 | 45.50 |
| MPEG1 | AK074166 | mRNA for FLJ00239 protein | 0.728 | 0.008465 | 0.566 | 36.14 |
| MS4A6A | NM_022349 | membrane-spanning 4-domains, subfamily A, member 6A | 0.793 | 0.00489715 | 1.838 | 64.76 |
| FCN1 | NM_002003 | ficolin (collagen/fibrinogen domain containing) 1 | 0.843 | 0.00801209 | 0.677 | 40.37 |
|  |  |  |  |  |  |  |
| ***Underexpressed genes after intake of olive oil phenols in men*** | | | | |  |  |
| ***Gene*** | ***GenBankID*** | ***Description*** | ***M-value*** | ***p-value*** | ***B-value*** | ***B-probability*** |
| ***Cytokines*** | |  |  |  |  |  |
| IL1B | NM_000576 | interleukin 1, beta | -1.599 | 0.00941587 | 0.346 | 25.71 |
| IL6 | NM_000600 | interleukin 6 | -0.997 | 0.00142772 | 6.532 | 86.72 |
| PBEF1 (X) | NM_005746 | pre-B-cell colony enhancing factor 1 | -0.856 | 0.00651644 | 1.126 | 52.96 |
| OSM | NM_020530 | oncostatin M | -0.700 | 0.00256245 | 3.342 | 76.97 |
| CMTM5 | NM_001037288 | CKLF-like MARVEL transmembrane domain containing 5 | -0.475 | 0.00181768 | 4.503 | 81.83 |
| IL1F8 | NM_173178 | interleukin 1 family, member 8 | -0.400 | 0.00783408 | 0.748 | 42.79 |
| ***Cytokine ligand*** | |  |  |  |  |  |
| CXCL2 (X) | NM_002089 | chemokine (C-X-C motif) ligand 2 | -0.785 | 0.00985872 | 0.232 | 18.83 |
| CXCL3 (X) | NM_002090 | chemokine (C-X-C motif) ligand 3 | -0.555 | 0.00262376 | 3.271 | 76.59 |
| ***Cytokine receptor*** | |  |  |  |  |  |
| CXCR4 (X) | NM_001008540 | chemokine (C-X-C motif) receptor 4 | -0.875 | 0.00156564 | 6.003 | 85.72 |
| ***Transporter*** | |  |  |  |  |  |
| SLC16A3 | AF318321 | pp10472 mRNA | -0.825 | 0.0084491 | 0.574 | 36.47 |
| LCN2 | NM_005564 | lipocalin 2 | -0.615 | 0.00906236 | 0.421 | 29.63 |
| ABCB9 | NM_019625 | ATP-binding cassette, sub-family B (MDR/TAP), member 9 | -0.569 | 0.00156564 | 5.844 | 85.39 |
| ATP9A | NM_006045 | ATPase, Class II, type 9A | -0.470 | 0.00592524 | 1.335 | 57.17 |
| SLC8A3 | NM_183002 | solute carrier family 8 (sodium-calcium exchanger), member 3 | -0.421 | 0.00518765 | 1.673 | 62.59 |
| SLC25A23 | NM_024103 | solute carrier family 25 | -0.420 | 0.00240731 | 3.589 | 78.21 |
| XK | NM_021083 | X-linked Kx blood group | -0.417 | 0.00408003 | 2.239 | 69.13 |
| ***Transmembrane receptor*** | |  |  |  |  |  |
| CD69 | NM_001781 | CD69 molecule | -0.927 | 0.0024266 | 3.512 | 77.84 |
| TAS2R50 (X) | NM_176890 | taste receptor, type 2, member 50 | -0.627 | 0.00448559 | 2.03 | 67.00 |
| IL7R | NM_002185 | interleukin 7 receptor | -0.474 | 0.00178952 | 4.621 | 82.21 |
| ITGB3 | NM_000212 | integrin, beta 3 | -0.444 | 0.0052962 | 1.62 | 61.83 |
| ***Ion Channel*** |  |  |  |  |  |  |
| CACNA1I | NM_021096 | calcium channel, voltage-dependent, alpha 1 | -0.503 | 0.00289409 | 2.993 | 74.96 |
| ***G-protein coupled receptor*** | |  |  |  |  |  |
| RGS1 | NM_002922 | regulator of G-protein signalling 1 | -1.169 | 0.0035035 | 2.523 | 71.62 |
| GNRHR | NM_000406 | gonadotropin-releasing hormone receptor | -0.547 | 0.00158767 | 5.794 | 85.28 |
| TBXA2R | NM_201636 | thromboxane A2 receptor | -0.537 | 0.00537419 | 1.582 | 61.27 |
| OR5L2 | NM_001004739 | olfactory receptor, family 5, subfamily L, member 2 | -0.520 | 0.0049043 | 1.83 | 64.66 |
| GP1BA | J02940 | Human platelet glycoprotein Ib alpha chain | -0.481 | 0.00918151 | 0.398 | 28.47 |
| MC1R | NM_002386 | melanocortin 1 receptor (alpha melanocyte stimulating hormone receptor) | -0.467 | 0.00676936 | 1.066 | 51.60 |
| ***Membrane proteins*** | |  |  |  |  |  |
| CD83 | NM_004233 | CD83 molecule | -1.595 | 0.00026173 | 4.255 | 80.97 |
| TMEM88 (X) | NM_203411 | transmembrane protein 88 | -1.267 | 0.00065001 | 2.69 | 72.90 |
| PCDH8 | NM_002590 | protocadherin 8 | -0.811 | 0.00768142 | 0.935 | 48.32 |
| LMOD1 (X) | NM_012134 | leiomodin 1 | -0.697 | 0.00934215 | 0.364 | 26.69 |
| NGFRAP1 | NM_014380 | nerve growth factor receptor | -0.550 | 0.00710843 | 0.958 | 48.93 |
| MMD | NM_012329 | monocyte to macrophage differentiation-associated | -0.540 | 0.00781941 | 0.754 | 42.99 |
| CLDN5 | NM_003277 | claudin 5 | -0.496 | 0.00672359 | 1.08 | 51.92 |
| ITGA2B | NM_000419 | integrin, alpha 2b | -0.458 | 0.0024266 | 3.504 | 77.80 |
| RTN4RL2 | NM_178570 | reticulon 4 receptor-like 2 | -0.423 | 0.00881657 | 0.477 | 32.30 |
| PARD3 | NM_019619 | par-3 partitioning defective 3 homolog (C. elegans) | -0.414 | 0.00365714 | 2.413 | 70.70 |
| NEXN | NM_144573 | nexilin | -0.414 | 0.00645855 | 1.149 | 53.47 |
| ***Enzymes*** | |  |  |  |  |  |
| PLA2G1B (X) | NM_000928 | phospholipase A2 | -0.564 | 0.00948259 | 0.332 | 24.92 |
| MSRA | NM_012331 | methionine sulfoxide reductase A | -0.540 | 0.00303334 | 2.847 | 74.01 |
| UBE2C | NM_181803 | ubiquitin-conjugating enzyme E2C | -0.523 | 0.00173895 | 5.334 | 84.21 |
| ALOX12 | NM_000697 | arachidonate 12-lipoxygenase | -0.515 | 0.00527574 | 1.631 | 61.99 |
| DNM3 | NM_015569 | dynamin 3 | -0.511 | 0.00870493 | 0.507 | 33.64 |
| SMOX | NM_175839 | spermine oxidase | -0.507 | 0.00616463 | 1.254 | 55.63 |
| GNG8 | NM_033258 | guanine nucleotide binding protein (G protein), gamma 8 | -0.494 | 0.00488562 | 1.843 | 64.83 |
| CML2 | NM_016347 | putative N-acetyltransferase Camello 2 | -0.480 | 0.00539106 | 1.574 | 61.15 |
| NTNG2 | NM_032536 | netrin G2 | -0.438 | 0.00646112 | 1.138 | 53.23 |
| SOD2 | NM_000636 | superoxide dismutase 2, mitochondrial | -0.433 | 0.00027426 | 4.153 | 80.59 |
| HERC2 | NM_004667 | hect domain and RLD 2 | -0.406 | 0.00788854 | 0.727 | 42.10 |
| ***Phosphatases*** | |  |  |  |  |  |
| PHACTR1 | AB051520 | mRNA for KIAA1733 protein | -0.897 | 0.00340925 | 2.572 | 72.00 |
| DUSP2 (X) | NM_004418 | dual specificity phosphatase 2 | -0.855 | 0.00178952 | 4.627 | 82.23 |
| PPP1R15A (X) | NM_014330 | protein phosphatase 1, regulatory (inhibitor) subunit 15A | -0.736 | 0.00449672 | 2.015 | 66.83 |
| PPP1R14A | NM_033256 | protein phosphatase 1, regulatory (inhibitor) subunit 14A | -0.531 | 0.00184128 | 4.374 | 81.39 |
| NT5C3 | NM_016489 | 5'-nucleotidase, cytosolic III | -0.469 | 0.00772292 | 0.779 | 43.79 |
| NT5M | NM_020201 | 5',3'-nucleotidase, mitochondrial | -0.431 | 0.00156564 | 5.89 | 85.49 |
| DOT1L | NM_032482 | DOT1-like, histone H3 methyltransferase | -0.417 | 0.003106 | 2.802 | 73.70 |
| ***Kinases*** | |  |  |  |  |  |
| SNF1LK | NM_173354 | SNF1-like kinase | -0.567 | 0.008465 | 0.569 | 36.27 |
| MYLK | NM_053025 | myosin, light polypeptide kinase | -0.513 | 0.0059782 | 1.319 | 56.88 |
| PIM3 | NM_001001852 | pim-3 oncogene | -0.476 | 0.00755723 | 0.841 | 45.68 |
| DMPK | NM_004409 | dystrophia myotonica-protein kinase | -0.450 | 0.00754295 | 0.849 | 45.92 |
| ***Metal ion binding*** | |  |  |  |  |  |
| LIMS3 (X) | NM_033514 | LIM and senescent cell antigen-like domains 3 | -0.995 | 0.00013925 | 5.348 | 84.25 |
| PVALB (X) | NM_002854 | parvalbumin | -0.635 | 0.00249193 | 3.422 | 77.39 |
| TNNC2 (X) | NM_003279 | troponin C type 2 | -0.620 | 0.00492159 | 1.805 | 64.35 |
| ***Protein Binding*** | |  |  |  |  |  |
| PER1 | NM_002616 | period homolog 1 (Drosophila) | -0.858 | 0.00269083 | 3.223 | 76.32 |
| APR-2 (X) | BC069097 | apoptosis related protein | -0.603 | 0.00658857 | 1.111 | 52.63 |
| ***Ligand-dependent nuclear receptor*** | | |  |  |  |  |
| NR4A1 | NM_002135 | nuclear receptor subfamily 4, group A, member 1 | -1.855 | 0.0000538 | 7.179 | 87.77 |
| AR | NM_000044 | androgen receptor | -0.495 | 0.00191727 | 4.145 | 80.56 |
| ***Signal trasduction*** | |  |  |  |  |  |
| TAGAP (X) | NM_138810 | T-cell activation GTPase activating protein | -0.904 | 0.000001 | 12.968 | 92.84 |
| SDCBP (X) | AK128645 | highly similar to syndecan binding protein | -0.727 | 0.00775194 | 0.767 | 43.41 |
| ***Signaling*** | |  |  |  |  |  |
| SOCS3 (X) | NM_003955 | suppressor of cytokine signaling 3 | -0.806 | 0.00178952 | 4.816 | 82.81 |
| NFKBIZ (X) | NM_031419 | nuclear factor of kappa light polypeptide gene enhancer in B-cells inhibitor, zeta | -0.470 | 0.00655451 | 1.119 | 52.81 |
| ***Cell cycle regulator*** | |  |  |  |  |  |
| G0S2 | NM_015714 | G0/G1switch 2 | -2.161 | 0.00575393 | 1.419 | 58.66 |
| CDKN2A (X) | NM_058197 | cyclin-dependent kinase inhibitor 2A | -0.884 | 0.00019275 | 4.728 | 82.54 |
| CDCA3 | NM_031299 | cell division cycle associated 3 | -0.454 | 0.00160984 | 5.658 | 84.98 |
| ***Translation regulator*** | |  |  |  |  |  |
| IGF2BP3 | NM_006547 | insulin-like growth factor 2 mRNA binding protein 3 | -0.451 | 0.00732939 | 0.898 | 47.31 |
| ***Transcription Regulator*** | |  |  |  |  |  |
| EGR1 (X) | NM_001964 | early growth response 1 | -1.590 | 0.00734961 | 0.891 | 47.12 |
| JUN (X) | NM_002228 | v-jun sarcoma virus 17 oncogene homolog (avian) | -1.294 | 0.00178952 | 4.746 | 82.60 |
| KLF6 (X) | NM_001008490 | Kruppel-like factor 6 | -0.692 | 0.00973041 | 0.261 | 20.70 |
| TRIM29 (X) | NM_012101 | tripartite motif-containing 29 | -0.677 | 0.00473631 | 1.902 | 65.54 |
| HTLF | NM_002158 | human T-cell leukemia virus enhancer factor | -0.675 | 0.00965619 | 0.276 | 21.63 |
| MLXIPL (X) | NM_032954 | MLX interacting protein-like, transcript variant 4 | -0.641 | 0.00962887 | 0.288 | 22.36 |
| JUNB (X) | NM_002229 | jun B proto-oncogene | -0.629 | 0.00272277 | 3.182 | 76.09 |
| HOXB13 (X) | NM_006361 | homeobox B13 | -0.578 | 0.00476307 | 1.894 | 65.45 |
| ZNF157 | NM_003446 | zinc finger protein 157 | -0.531 | 0.00550731 | 1.523 | 60.36 |
| NANOGP1 | AK097770 | cDNA FLJ40451 fis | -0.527 | 0.00965348 | 0.279 | 21.81 |
| ZFP36 | NM_003407 | zinc finger protein 36, C3H type, homolog (mouse) | -0.519 | 0.00673673 | 1.073 | 51.76 |
| MEIS1 | NM_002398 | Meis1, myeloid ecotropic viral integration site 1 homolog (mouse) | -0.497 | 0.00537649 | 1.58 | 61.24 |
| GFI1B | NM_004188 | growth factor independent 1B | -0.467 | 0.00178952 | 5.147 | 83.73 |
| ZNF628 (X) | NM_033113 | zinc finger protein 628 | -0.439 | 0.00178952 | 4.821 | 82.82 |
| HEXIM2 | NM_144608 | hexamthylene bis-acetamide inducible 2 | -0.418 | 0.00705724 | 0.977 | 49.42 |
| MAFF | NM_012323 | v-maf musculoaponeurotic fibrosarcoma oncogene homolog F (avian) | -0.405 | 0.00551539 | 1.51 | 60.16 |
| ***Hypothetical genes or non-annotated function genes*** | | |  |  |  |  |
| LOC284454 (X) | AL832183 | mRNA | -1.952 | 0.00026193 | 4.25 | 80.95 |
| LRRC41 (X) | AK024051 | highly similar to H.sapiens mRNA for MUF1 protein | -0.771 | 0.00601792 | 1.304 | 56.60 |
| IGHG1 | NM_001040077 | anti-rabies SO57 immunoglobulin heavy chain | -0.746 | 0.00982806 | 0.244 | 19.61 |
| MPEG1 | AK074166 | mRNA for FLJ00239 protein | 0.728 | 0.008465 | 0.566 | 36.14 |
| FLJ11235 | AK002097 | cDNA FLJ11235 fis | -0.674 | 0.00580878 | 1.396 | 58.26 |
| SKI | NM_003036 | v-ski sarcoma viral oncogene homolog (avian) | -0.659 | 0.00876112 | 0.496 | 33.16 |
| C19orf19 (X) | NM_182577 | chromosome 19 open reading frame 19 | -0.654 | 0.00950686 | 0.326 | 24.59 |
| IER2 (X) | NM_004907 | immediate early response 2 | -0.651 | 0.00160984 | 5.627 | 84.91 |
| PACAP | NM_016459 | proapoptotic caspase adaptor protein | -0.612 | 0.00598379 | 1.315 | 56.80 |
| KIAA1922 | BC033082 | KIAA1922 protein | -0.612 | 0.00992531 | 0.217 | 17.83 |
| ATXN7L2 | NM_153340 | ataxin 7-like 2 | -0.592 | 0.00881039 | 0.483 | 32.57 |
| MOSPD2 | NM_152581 | motile sperm domain containing 2 | 0.563 | 0.00449672 | 2.013 | 66.81 |
| SH3BGRL2 | NM_031469 | SH3 domain binding glutamic acid-rich protein like 2 | -0.561 | 0.00615487 | 1.26 | 55.75 |
| FAM46C | NM_017709 | family with sequence similarity 46, member C | -0.556 | 0.00835765 | 0.592 | 37.19 |
| FLJ22795 | AF316855 | colon cancer-associated antigen AgSK1-2HT-ECS mRNA | -0.554 | 0.00485333 | 1.868 | 65.13 |
| C10orf63 | NM_145010 | chromosome 10 open reading frame 63 | -0.553 | 0.00982806 | 0.247 | 19.81 |
| SPOCD1 | NM_144569 | SPOC domain containing 1 | -0.529 | 0.00351637 | 2.495 | 71.39 |
| SCGB1C1 | NM_145651 | secretoglobin, family 1C, member 1 | -0.518 | 0.0072839 | 0.918 | 47.86 |
| CLU | NM_203339 | clusterin | -0.514 | 0.00434399 | 2.097 | 67.71 |
| NMU | NM_006681 | neuromedin U | -0.504 | 0.00627458 | 1.216 | 54.87 |
| TUBA8 | NM_018943 | tubulin, alpha 8 | -0.500 | 0.00881657 | 0.473 | 32.11 |
| BHLHB8 | BX648200 | mRNA; cDNA DKFZp779C0742 | -0.500 | 0.0050706 | 1.736 | 63.45 |
| HIST1H2AE | NM_021052 | histone 1, H2ae | -0.499 | 0.00594517 | 1.327 | 57.03 |
| OTUD6A | NM_207320 | OTU domain containing 6A | -0.499 | 0.00382804 | 2.326 | 69.93 |
| MYL9 | NM_181526 | myosin, light polypeptide 9, regulatory | -0.498 | 0.00181768 | 4.434 | 81.60 |
| ACRBP | NM_032489 | acrosin binding protein | -0.495 | 0.00455389 | 1.989 | 66.54 |
| C5orf4 | NM_032385 | chromosome 5 open reading frame 4 | -0.489 | 0.00550731 | 1.523 | 60.36 |
| PTCRA | NM_138296 | pre T-cell antigen receptor alpha | -0.486 | 0.00216183 | 3.815 | 79.23 |
| ZNF659 | NM_024697 | zinc finger protein 659 | -0.486 | 0.00662158 | 1.102 | 52.43 |
| BIRC5 | NM_001012271 | baculoviral IAP repeat-containing 5 (survivin) | -0.485 | 0.00214296 | 3.86 | 74.04 |
| CPNE5 | NM_020939 | copine V | -0.477 | 0.00191727 | 4.156 | 80.61 |
| FHL2 | NM_201555 | four and a half LIM domains 2 | -0.477 | 0.0083246 | 0.598 | 37.42 |
| U85992 | U85992 | unknown protein | -0.474 | 0.00571487 | 1.462 | 59.38 |
| PDZK1IP1 | NM_005764 | PDZK1 interacting protein 1 | -0.471 | 0.00272277 | 3.19 | 76.13 |
| HIST1H3B | NM_003537 | histone 1, H3b | -0.469 | 0.00191727 | 4.12 | 80.47 |
| SH3TC2 | NM_024577 | SH3 domain and tetratricopeptide repeats 2 | -0.466 | 0.00565091 | 1.469 | 59.50 |
| LPP | NM_005578 | LIM domain containing preferred translocation partner in lipoma | -0.463 | 0.00707865 | 0.972 | 49.29 |
| ADM | NM_001124 | adrenomedullin | -0.460 | 0.00443499 | 2.057 | 67.29 |
| FAM43B | NM_207334 | family with sequence similarity 43, member B | -0.456 | 0.008465 | 0.563 | 36.02 |
| SNCB | NM_001001502 | synuclein, beta | -0.455 | 0.00950836 | 0.318 | 24.13 |
| LOC133874 | BC092511 | cDNA clone IMAGE:5271968 | -0.455 | 0.00700539 | 1.002 | 50.05 |
| CCDC3 | NM_031455 | coiled-coil domain containing 3 | -0.454 | 0.00504236 | 1.763 | 63.81 |
| TMEM40 | NM_018306 | transmembrane protein 40 | -0.450 | 0.00492159 | 1.806 | 64.36 |
| LOC541469 | NM_001013617 | hypothetical LOC541469 protein | -0.448 | 0.00434285 | 2.102 | 67.76 |
| HIST1H3D | NM_003530 | histone 1, H3d | -0.447 | 0.00275731 | 3.139 | 75.84 |
| TREML1 | AY358357 | unknown protein | -0.446 | 0.00646112 | 1.14 | 53.27 |
| KENAE | NM_176816 | Kenae | -0.443 | 0.00312193 | 2.783 | 73.57 |
| Ells1 | NM_152793 | hypothetical protein Ells1 | -0.441 | 0.008465 | 0.564 | 36.06 |
| C18orf1 | AK023474 | cDNA FLJ13412 fis, clone PLACE1001745 | -0.439 | 0.0061698 | 1.242 | 55.40 |
| IFI27 | NM_005532 | interferon, alpha-inducible protein 27 | -0.436 | 0.00956332 | 0.303 | 23.25 |
| TSPAN18 | NM_130783 | tetraspanin 18 | -0.434 | 0.00198119 | 4.009 | 80.04 |
| HIST1H3H | NM_003536 | histone 1, H3h | -0.434 | 0.00184128 | 4.383 | 81.42 |
| N59744 | N59744 | N59744 yv56g04.r1 Soares fetal liver spleen 1NFLS | -0.431 | 0.00626604 | 1.218 | 54.91 |
| HIST2H2AA3 | NM_003516 | histone 2, H2aa3 | -0.427 | 0.00216183 | 3.821 | 79.26 |
| CCNK | NM_003858 | cyclin K | -0.426 | 0.0059816 | 1.318 | 56.86 |
| HIST2H2BE | NM_003528 | histone 2, H2be | -0.426 | 0.00178952 | 4.901 | 83.05 |
| OVOS2 | BC039117 | ovostatin 2 | -0.422 | 0.00995623 | 0.207 | 17.15 |
| HIST1H2BH | NM_003524 | histone 1, H2bh | -0.421 | 0.00322476 | 2.724 | 73.15 |
| SELP | NM_003005 | selectin P | -0.419 | 0.00965619 | 0.277 | 21.69 |
| SAMD14 | NM_174920 | sterile alpha motif domain containing 14 | -0.417 | 0.00323095 | 2.677 | 72.80 |
| GAS2L1 | NM_152237 | growth arrest-specific 2 like 1 | -0.416 | 0.00463462 | 1.947 | 66.07 |
| HIST1H2BO | NM_003527 | histone 1, H2bo | -0.413 | 0.00225853 | 3.683 | 78.65 |
| RGS10 | NM_001005339 | regulator of G-protein signalling 10 | -0.413 | 0.00470287 | 1.914 | 65.68 |
| C10orf47 (X) | NM_153256 | chromosome 10 open reading frame 47 | -0.410 | 0.00449422 | 2.024 | 66.93 |
| TP53TG3 | NM_016212 | TP53TG3 protein | -0.407 | 0.0099358 | 0.211 | 17.42 |
| FSTL1 | NM_007085 | follistatin-like 1 | -0.403 | 0.00247202 | 3.467 | 77.61 |
| ProSAPiP1 | NM_014731 | ProSAPiP1 protein | -0.402 | 0.00755723 | 0.843 | 45.74 |
|  |  |  |  |  |  |  |
| ***Overexpressed genes after intake of olive oil phenols in women*** | | | | |  |  |
| ***Gene*** | ***GenBankID*** | ***Description*** | ***M-value*** | ***p-value*** | ***B-value*** | ***B-probability*** |
| ***Cytokine*** |  |  |  |  |  |  |
| FASLG | NM_000639 | Fas ligand (TNF superfamily, member 6) | 0.453 | 0.0048901 | 1.342 | 57.30 |
| ***Transporter*** |  |  |  |  |  |  |
| ABCC13 | NR_003088 | ATP-binding cassette, sub-family C (CFTR/MRP), member 13 | 0.849 | 0.0014718 | 3.368 | 77.11 |
| SLC4A1 | NM_000342 | solute carrier family 4, anion exchanger, member 1 | 0.535 | 0.00078854 | 4.433 | 81.59 |
| ***Membrane Protein*** | |  |  |  |  |  |
| GYPB | NM_002100 | glycophorin B | 0.961 | 0.00240542 | 2.567 | 71.97 |
| ***Metal ion binding*** |  |  |  |  |  |  |
| SELENBP1 | NM_003944 | selenium binding protein 1 | 0.783 | 0.00056271 | 4.978 | 83.27 |
| ***Kinase*** |  |  |  |  |  |  |
| ALS2CR2 | NM_018571 | amyotrophic lateral sclerosis 2 (juvenile) chromosome region, candidate 2 | 0.520 | 0.00343464 | 1.994 | 66.60 |
| ***Enzymes*** |  |  |  |  |  |  |
| RSAD2 | NM_080657 | radical S-adenosyl methionine domain containing 2 | 0.417 | 0.00442435 | 1.548 | 60.75 |
| GSPT1 | NM_002094 | G1 to S phase transition 1 | 0.445 | 0.00155242 | 3.259 | 76.52 |
| CD38 | NM_001775 | CD38 molecule | 0.446 | 0.00856035 | 0.41 | 29.08 |
| TMOD1 | NM_003275 | tropomodulin 1 | 0.447 | 0.00168417 | 3.122 | 75.74 |
| FECH | NM_001012515 | ferrochelatase (protoporphyria) | 0.567 | 0.0007301 | 4.601 | 82.15 |
| CA1 | NM_001738 | carbonic anhydrase I | 0.858 | 0.00790765 | 0.555 | 35.69 |
| ***Transcription Regulator*** | |  |  |  |  |  |
| HOXA10 | NM_018951 | homeobox A10 | 0.415 | 0.00999546 | 0.164 | 14.09 |
| ***Hypothetical genes or non-annotated function genes*** | | |  |  |  |  |
| FLJ22746 | NM_024785 | hypothetical protein FLJ22746 | 0.406 | 0.00732529 | 0.702 | 41.25 |
| FANCF | NM_022725 | Fanconi anemia, complementation group F | 0.409 | 0.00285093 | 2.28 | 69.51 |
| L06610 | L06610 | Human rearranged immunoglobulin heavy chain | 0.412 | 0.00849321 | 0.432 | 30.17 |
| LOC63920 | NM_022090 | transposon-derived Buster3 transposase-like | 0.428 | 0.00856035 | 0.41 | 29.08 |
| ZNF702 | NM_024924 | zinc finger protein 702 | 0.471 | 0.00318467 | 2.102 | 67.76 |
| SCUBE2 | NM_020974 | signal peptide, CUB domain, EGF-like 2 | 0.472 | 0.00168417 | 3.145 | 75.87 |
| TMEM56 | NM_152487 | transmembrane protein 56 | 0.493 | 0.00826925 | 0.502 | 33.42 |
| WDR40A | NM_015397 | WD repeat domain 40A | 0.518 | 0.00382138 | 1.803 | 64.32 |
| IFIT1 | NM_001548 | interferon-induced protein with tetratricopeptide repeats 1 | 0.544 | 0.00183031 | 2.974 | 74.84 |
| PEX12 | NM_000286 | peroxisomal biogenesis factor 12 | 0.549 | 0.00387979 | 1.782 | 64.05 |
| LOC389599 | XM_372002 | PREDICTED: similar to amyotrophic lateral sclerosis 2 | 0.585 | 0.00751193 | 0.655 | 39.58 |
| LOC114227 | AF130049 | clone FLB3411 PRO0852 mRNA | 0.933 | 0.0066996 | 1.017 | 50.42 |
|  |  |  |  |  |  |  |
| ***Underexpressed genes after intake of olive oil phenols in women*** | | | | |  |  |
| ***Gene*** | ***GenBankID*** | ***Description*** | ***M-value*** | ***p-value*** | ***B-value*** | ***B-probability*** |
| ***Cytokines*** |  |  |  |  |  |  |
| PBEF1 (X) | NM_005746 | pre-B-cell colony enhancing factor 1 | -1.063 | 0.00181501 | 3.005 | 75.03 |
| SPP1 | NM_000582 | secreted phosphoprotein 1 | -0.612 | 0.00475445 | 1.484 | 59.74 |
| PF4 | NM_002619 | platelet factor 4 (chemokine (C-X-C motif) ligand 4) | -0.540 | 0.00180032 | 3.032 | 75.20 |
| PF4V1 | NM_002620 | platelet factor 4 variant 1 | -0.406 | 0.00129443 | 3.586 | 78.19 |
| ***Growth factor*** | |  |  |  |  |  |
| EREG | NM_001432 | epiregulin | -1.365 | 0.0095522 | 0.241 | 19.42 |
| PDGFA | NM_002607 | platelet-derived growth factor alpha polypeptide | -0.403 | 0.00057278 | 4.955 | 83.21 |
| ***Cytokine ligand*** | |  |  |  |  |  |
| CXCL1 | NM_001511 | chemokine (C-X-C motif) ligand 1 | -1.733 | 0.00073366 | 4.585 | 82.09 |
| CXCL2 (X) | NM_002089 | chemokine (C-X-C motif) ligand 2 | -0.793 | 0.0043412 | 1.586 | 61.33 |
| CXCL5 | NM_002994 | chemokine (C-X-C motif) ligand 5 | -0.413 | 0.00129443 | 3.584 | 78.18 |
| CXCL3 (X) | NM_002090 | chemokine (C-X-C motif) ligand 3 | -0.404 | 0.00006952 | 8.447 | 89.41 |
| ***Cytokine receptor*** | |  |  |  |  |  |
| CXCR4 (X) | NM_001008540 | chemokine (C-X-C motif) receptor 4 | -0.708 | 0.00434557 | 1.578 | 61.21 |
| ***Transporter*** |  |  |  |  |  |  |
| KIF13A | NM_022113 | kinesin family member 13A | -0.504 | 0.00659573 | 0.884 | 46.92 |
| SLC24A3 | NM_020689 | solute carrier family 24 | -0.424 | 0.00006952 | 8.309 | 89.26 |
| ***Transmembrane receptor*** | |  |  |  |  |  |
| IL1R2 | NM_004633 | interleukin 1 receptor, type II | -0.702 | 0.00255254 | 2.452 | 71.03 |
| TAS2R50 (X) | NM_176890 | taste receptor, type 2, member 50 | -0.564 | 0.00017005 | 7.058 | 87.59 |
| IL6ST | U58146 | alternatively spliced interleukin-6 receptor beta chain | -0.467 | 0.00422986 | 1.631 | 61.99 |
| ***Ion Channel*** |  |  |  |  |  |  |
| KCNV2 | NM_133497 | potassium channel, subfamily V, member 2 | -0.505 | 0.00019359 | 6.728 | 87.06 |
| ***Membrane Protein*** | |  |  |  |  |  |
| FCAR | NM_133280 | Fc fragment of IgA, receptor for (FCAR), transcript variant 10 | -0.965 | 0.00257173 | 2.439 | 70.92 |
| TMEM88 (X) | NM_203411 | transmembrane protein 88 | -0.625 | 0.00777129 | 0.591 | 37.15 |
| LMOD1 (X) | NM_012134 | leiomodin 1 (smooth muscle) | -0.621 | 0.00001768 | 11.542 | 92.03 |
| DOCK4 | AK055497 | cDNA FLJ30935 fis | -0.586 | 0.00384781 | 1.795 | 64.22 |
| SPAG9 | BC007524 | sperm associated antigen 9 | -0.546 | 0.00766176 | 0.619 | 38.23 |
| SDPR | NM_004657 | serum deprivation response (phosphatidylserine binding protein) | -0.522 | 0.00048107 | 5.233 | 83.96 |
| EMP1 | BC017854 | epithelial membrane protein 1 | -0.466 | 0.00481908 | 1.388 | 58.12 |
| ***Enzymes*** |  |  |  |  |  |  |
| PTGS2 | NM_000963 | prostaglandin-endoperoxide synthase 2 | -1.892 | 0.00076798 | 4.495 | 81.80 |
| SOD2 | BC016934 | superoxide dismutase 2, mitochondrial | -1.292 | 0.00221846 | 2.676 | 72.80 |
| KRT23 | NM_015515 | keratin 23 (histone deacetylase inducible) | -0.828 | 0.00265902 | 2.39 | 70.50 |
| THEM5 | NM_182578 | thioesterase superfamily member 5 | -0.612 | 0.00002874 | 10.056 | 90.96 |
| GNG11 | NM_004126 | guanine nucleotide binding protein (G protein), gamma 11 | -0.579 | 0.00032544 | 5.76 | 85.21 |
| VNN3 | NM_018399 | vanin 3 (VNN3), transcript variant 1 | -0.553 | 0.00493568 | 1.325 | 56.99 |
| IDS | NM_006123 | iduronate 2-sulfatase (Hunter syndrome) | -0.497 | 0.0043314 | 1.591 | 61.40 |
| PLA2G1B (X) | NM_000928 | phospholipase A2, group IB | -0.491 | 0.0002568 | 6.149 | 86.01 |
| CYBA | BC028224 | cytochrome b-245, alpha polypeptide | -0.482 | 0.00177683 | 3.049 | 75.30 |
| GNAZ | NM_002073 | guanine nucleotide binding protein (G protein), alpha z polypeptide | -0.466 | 0.00002874 | 9.822 | 90.76 |
| CA2 | NM_000067 | carbonic anhydrase II | -0.465 | 0.00021849 | 6.526 | 86.71 |
| LYCAT | AK095284 | cDNA FLJ37965 fis | -0.464 | 0.0095324 | 0.244 | 19.61 |
| TGM4 | NM_003241 | transglutaminase 4 (prostate) | -0.461 | 0.00021463 | 6.592 | 86.83 |
| HGD | NM_000187 | homogentisate 1,2-dioxygenase (homogentisate oxidase) | -0.457 | 0.00001268 | 12.545 | 92.62 |
| ANXA3 | NM_005139 | annexin A3 | -0.451 | 0.00087206 | 4.253 | 80.96 |
| HSD17B3 | NM_000197 | hydroxysteroid (17-beta) dehydrogenase 3 | -0.428 | 0.00570687 | 1.095 | 52.27 |
| SAT | NM_002970 | spermidine/spermine N1-acetyltransferase | -0.420 | 0.00352315 | 1.952 | 66.12 |
| GSTA2 | NM_000846 | glutathione S-transferase A2 | -0.413 | 0.00849093 | 0.433 | 30.22 |
| F13A1 | NM_000129 | coagulation factor XIII, A1 polypeptide | -0.407 | 0.00059044 | 4.909 | 83.08 |
| ***Phosphatases*** | |  |  |  |  |  |
| DUSP1 | NM_004417 | dual specificity phosphatase 1 | -1.216 | 0.00343927 | 1.99 | 66.56 |
| DUSP2 (X) | NM_004418 | dual specificity phosphatase 2 | -0.762 | 0.00735717 | 0.695 | 41.00 |
| PPP1R15A (X) | NM_014330 | protein phosphatase 1, regulatory (inhibitor) subunit 15A | -0.682 | 0.00545745 | 1.159 | 53.68 |
| PTPRG | NM_002841 | protein tyrosine phosphatase, receptor type, G | -0.473 | 0.00356669 | 1.922 | 65.78 |
| SSH2 | AB072358 | mRNA for hSSH-2 | -0.404 | 0.00149031 | 3.322 | 76.86 |
| ***Kinases*** |  |  |  |  |  |  |
| SGK | NM_005627 | serum/glucocorticoid regulated kinase | -1.097 | 0.00031296 | 5.822 | 85.34 |
| TRIB1 | NM_025195 | tribbles homolog 1 (Drosophila) | -0.750 | 0.00790765 | 0.554 | 35.65 |
| ***Metal ion binding*** |  |  |  |  |  |  |
| LIMS3 (X) | NM_033514 | LIM and senescent cell antigen-like domains 3 | -0.555 | 0.00005257 | 8.942 | 89.94 |
| TNNC2 (X) | NM_003279 | troponin C type 2 (fast) | -0.519 | 0.00003164 | 9.545 | 90.52 |
| PVALB (X) | NM_002854 | parvalbumin | -0.445 | 0.00008381 | 8.009 | 88.90 |
| ***Protein Binding*** | |  |  |  |  |  |
| NPHS2 | NM_014625 | nephrosis 2, idiopathic, steroid-resistant (podocin) | -0.653 | 0.00008126 | 8.061 | 88.96 |
| APR-2 (X) | BC069097 | apoptosis related protein | -0.526 | 0.00002874 | 10.069 | 90.97 |
| ***Signal trasduction*** | |  |  |  |  |  |
| SDCBP (X) | AK128645 | cDNA FLJ46804 fis | -0.687 | 0.00850034 | 0.429 | 30.02 |
| TAGAP (X) | NM_138810 | T-cell activation GTPase activating protein | -0.567 | 0.00146213 | 3.405 | 77.30 |
| ***Signaling*** | |  |  |  |  |  |
| NFKBIZ (X) | NM_031419 | nuclear factor of kappa light polypeptide gene enhancer in B-cells inhibitor, zeta (NFKBIZ), transcript variant 1, mRNA [NM_031419] | -0.843 | 0.00662083 | 0.876 | 46.70 |
| SOCS3 (X) | NM_003955 | suppressor of cytokine signaling 3 | -0.806 | 0.00437153 | 1.571 | 61.10 |
| ***Cell cycle regulator*** | |  |  |  |  |  |
| CDKN2A (X) | NM_058197 | cyclin-dependent kinase inhibitor 2A | -0.438 | 0.00002874 | 9.833 | 90.77 |
| ***Ligand-dependent nuclear receptor*** | | |  |  |  |  |
| NR4A2 (X) | NM_006186 | nuclear receptor subfamily 4, group A, member 2 | -1.843 | 0.00187162 | 2.93 | 74.55 |
| ***Transcription Regulator*** | |  |  |  |  |  |
| EGR2 | NM_000399 | early growth response 2 (Krox-20 homolog, Drosophila) | -2.361 | 0.00184324 | 2.953 | 85.31 |
| EGR1 (X) | NM_001964 | early growth response 1 | -2.322 | 0.00031534 | 5.807 | 70.67 |
| FOSB | NM_006732 | FBJ murine osteosarcoma viral oncogene homolog B | -2.220 | 0.00078854 | 4.442 | 81.62 |
| EGR3 | NM_004430 | early growth response 3 | -1.722 | 0.00268049 | 2.409 | 74.70 |
| JUN (X) | NM_002228 | v-jun sarcoma virus 17 oncogene homolog (avian) | -1.069 | 0.00821836 | 0.487 | 32.75 |
| KLF6 (X) | NM_001008490 | Kruppel-like factor 6 | -0.783 | 0.00849093 | 0.433 | 30.22 |
| JUNB (X) | NM_002229 | jun B proto-oncogene | -0.768 | 0.00297483 | 2.225 | 68.99 |
| ZFP36 (X) | NM_003407 | zinc finger protein 36, C3H type, homolog (mouse) | -0.699 | 0.00485065 | 1.365 | 57.72 |
| MXD1 | NM_002357 | MAX dimerization protein 1 | -0.642 | 0.0007417 | 4.551 | 81.99 |
| TRIM29 (X) | NM_012101 | tripartite motif-containing 29 | -0.587 | 0.00002874 | 9.929 | 90.85 |
| MLXIPL (X) | NM_032954 | MLX interacting protein-like (MLXIPL), transcript variant 4 | -0.531 | 0.00002662 | 10.546 | 91.34 |
| HOXB13 (X) | NM_006361 | homeobox B13 | -0.467 | 0.00017979 | 6.966 | 87.45 |
| ETV3 | NM_005240 | ets variant gene 3 | -0.447 | 0.00979189 | 0.198 | 16.53 |
| ***Hypothetical genes or non-annotated function genes*** | | |  |  |  |  |
| PRG1 | BC022313 | proteoglycan 1, secretory granule | -1.012 | 0.00813684 | 0.504 | 33.51 |
| LOC284454 (X) | AL832183 | mRNA; cDNA DKFZp686D0720 | -1.010 | 0.00652555 | 0.902 | 47.42 |
| FLJ22659 | AK026312 | cDNA: FLJ22659 fis | -0.904 | 0.00754067 | 0.65 | 39.39 |
| LIX1 | NM_153234 | Lix1 homolog (mouse) | -0.792 | 0.00406744 | 1.824 | 64.59 |
| LOC389607 | NM_001013651 | hypothetical gene supported by AK128318 | -0.691 | 0.0004335 | 5.358 | 84.27 |
| IER2 (X) | NM_004907 | immediate early response 2 | -0.678 | 0.00381621 | 1.807 | 64.37 |
| AY029066 | AY029066 | Humanin (HN1) mRNA, complete cds. [AY029066] | -0.666 | 0.00060123 | 4.886 | 83.01 |
| LOC399900 | NM_001013667 | hypothetical gene supported by AK093779 | -0.626 | 0.00320545 | 2.093 | 67.67 |
| LRRC41 (X) | AK024051 | cDNA FLJ13989 fis | -0.613 | 0.00002662 | 10.438 | 91.26 |
| FLJ11235 | AK002097 | cDNA FLJ11235 fis | -0.600 | 0.00001768 | 11.381 | 91.92 |
| TFF1 | NM_003225 | trefoil factor 1 | -0.586 | 0.00001768 | 11.09 | 91.73 |
| PMAIP1 | NM_021127 | phorbol-12-myristate-13-acetate-induced protein 1 | -0.564 | 0.00308806 | 2.157 | 68.32 |
| RGS18 | NM_130782 | regulator of G-protein signalling 18 | -0.550 | 0.00019359 | 6.751 | 87.10 |
| FAM81B | NM_152548 | family with sequence similarity 81, member B | -0.543 | 0.00010907 | 7.617 | 88.40 |
| C9orf47 | NM_001001938 | chromosome 9 open reading frame 47 | -0.540 | 0.00165985 | 3.158 | 75.95 |
| ATXN7L2 | NM_153340 | ataxin 7-like 2 (ATXN7L2), mRNA [NM_153340] | -0.539 | 0.00002662 | 10.384 | 91.22 |
| C10orf63 (X) | NM_145010 | chromosome 10 open reading frame 63 (C10orf63), mRNA [NM_145010] | -0.533 | 0.00020499 | 6.648 | 86.92 |
| LOC646686 | AK097297 | cDNA FLJ39978 fis | -0.532 | 0.00574445 | 1.083 | 51.99 |
| C19orf19 (X) | NM_182577 | chromosome 19 open reading frame 19 (C19orf19), mRNA [NM_182577] | -0.526 | 0.00025187 | 6.186 | 86.08 |
| DENND2C | NM_198459 | DENN/MADD domain containing 2C | -0.517 | 0.00007186 | 8.224 | 89.16 |
| C21orf7 | NM_020152 | chromosome 21 open reading frame 7 (C21orf7), mRNA [NM_020152] | -0.514 | 0.00135347 | 3.524 | 77.90 |
| DNHD2 | NM_178504 | dynein heavy chain domain 2 | -0.503 | 0.00032544 | 5.753 | 85.19 |
| LOC90834 | BC001742 | Homo sapiens, clone IMAGE:3535910 | -0.503 | 0.00600396 | 1.015 | 50.37 |
| TFPI | NM_006287 | tissue factor pathway inhibitor | -0.502 | 0.00005637 | 8.839 | 89.84 |
| KIAA1922 | BC033082 | KIAA1922 protein | -0.498 | 0.00021727 | 6.556 | 86.77 |
| MALAT1 | NR_002819 | metastasis associated lung adenocarcinoma transcript 1 (non-coding RNA) | -0.478 | 0.00748088 | 0.663 | 39.87 |
| S81524 | S81524 | RC1=NADH dehydrogenase subunit 3 homolog/ND3 homolog | -0.471 | 0.00016215 | 7.154 | 87.74 |
| SPARC | NM_003118 | secreted protein, acidic, cysteine-rich (osteonectin) | -0.465 | 0.00067383 | 4.734 | 82.56 |
| MN1 | NM_002430 | meningioma (disrupted in balanced translocation) 1 | -0.464 | 0.00453166 | 1.499 | 59.98 |
| TMEM45A | NM_018004 | transmembrane protein 45A | -0.463 | 0.00343927 | 1.989 | 66.54 |
| TncRNA | U60873 | Human clone 137308 mRNA | -0.450 | 0.001061 | 3.907 | 79.62 |
| FRZB | NM_001463 | frizzled-related protein | -0.439 | 0.0076499 | 0.625 | 38.46 |
| HSPC159 | NM_014181 | HSPC159 protein | -0.439 | 0.00459875 | 1.472 | 59.55 |
| PRAC | NM_032391 | small nuclear protein PRAC | -0.437 | 0.00850034 | 0.424 | 29.78 |
| FLJ11903 | AK021965 | cDNA FLJ11903 fis | -0.436 | 0.00738789 | 0.69 | 40.83 |
| YPEL5 | NM_016061 | yippee-like 5 (Drosophila) | -0.431 | 0.00175605 | 3.072 | 75.44 |
| LOC285944 | AK091933 | cDNA FLJ34614 fis | -0.430 | 0.00767596 | 0.61 | 37.89 |
| U2AF1 | AL832665 | mRNA; cDNA DKFZp313J1712 | -0.429 | 0.00255254 | 2.453 | 71.04 |
| PSG7 | NM_002783 | pregnancy specific beta-1-glycoprotein 7 | -0.427 | 0.00569171 | 1.104 | 52.47 |
| HRASLS | NM_020386 | HRAS-like suppressor | -0.420 | 0.0008813 | 4.239 | 80.91 |
| CCNL1 | NM_020307 | cyclin L1 (CCNL1) | -0.419 | 0.002809 | 2.298 | 69.68 |
| MFAP3L | NM_021647 | microfibrillar-associated protein 3-like (MFAP3L), transcript variant 1 | -0.417 | 0.00032969 | 5.71 | 85.10 |
| C15orf26 | NM_173528 | chromosome 15 open reading frame 26 | -0.409 | 0.00039966 | 5.492 | 84.60 |
| CR627362 | CR627362 | mRNA; cDNA DKFZp686H21113 | -0.403 | 0.00212923 | 2.739 | 73.25 |

M-values [log2(ratio)] expressing a fold change after high-phenol olive oil acute intake compared to low-phenol olive oil consumption. **X**: differentially expressed by olive oil phenols in the men and women analysis.
